# New metathesis catalyst bearing chromanyl moieties at the N-heterocyclic carbene ligand

**DOI:** 10.3762/bjoc.11.300

**Published:** 2015-12-30

**Authors:** Agnieszka Hryniewicka, Szymon Suchodolski, Agnieszka Wojtkielewicz, Jacek W Morzycki, Stanisław Witkowski

**Affiliations:** 1 University of Białystok, Institute of Chemistry, Ciołkowskiego Street 1K, 15-245 Białystok; Poland

**Keywords:** chromane derivatives, metathesis catalyst, nitrogen heterocycles, olefin metathesis, Ru-carbene

## Abstract

The synthesis of a new type of Hoveyda–Grubbs 2^nd^ generation catalyst bearing a modified N-heterocyclic carbene ligands is reported. The new catalyst contains an NHC ligand symmetrically substituted with chromanyl moieties. The complex was tested in model CM and RCM reactions. It showed very high activity in CM reactions with electron-deficient α,β-unsaturated compounds even at 0 °C. It was also examined in more demanding systems such as conjugated dienes and polyenes. The catalyst is stable, storable and easy to purify.

## Introduction

Olefin metathesis is still one of the most intensively studied transformations in synthetic organic chemistry. It has been frequently used as a key bond-forming reaction for total syntheses of many natural products [1]. The study on designing new metathesis catalysts and their synthesis has been a very fast developing area of organic chemistry since 1992, when Grubbs discovered the first well-defined ruthenium catalyst [2]. Nearly 400 ruthenium heterocyclic carbene-coordinated olefin metathesis catalysts were prepared until 2010 [3]. Since 2011, when Grubbs reported the synthesis of a *Z*-selective catalyst [4], several modified stereoselective catalysts were described [5–8]. Over the last few years, considerable attention has also been paid to immobilisation and tagging of catalysts and especially on making them more environmentally friendly [9–14].

**Figure 1:**
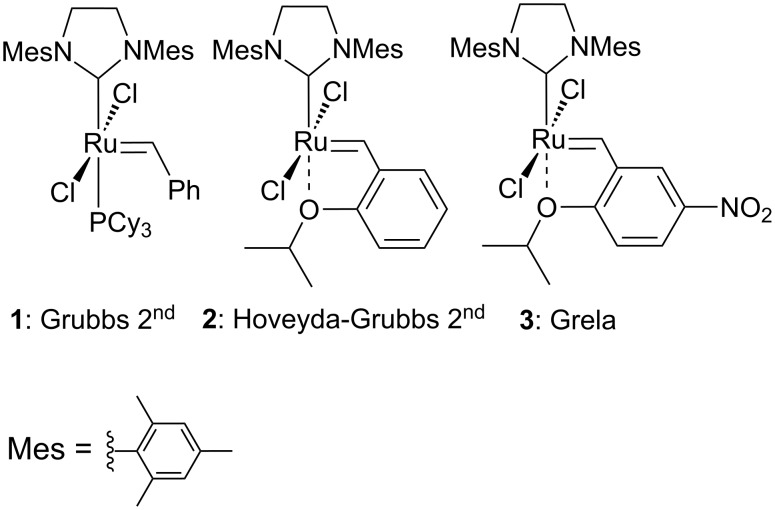
Examples of olefin metathesis ruthenium catalysts.

Alkene cross-metathesis (CM) is a convenient route to the synthesis of functionalised olefins from simple precursors. Since the discovery of Grubbs 2^nd^ generation catalyst (**1**, Figure 1) [15], Hoveyda–Grubbs 2^nd^ generation catalyst (**2**, Figure 1) [16] and some successful modifications, e.g., nitro-Grela catalyst (**3**, Figure 1) [17] the utility of CM has been continuously expanded. The synthesis of complex structures bearing polar functional groups can be accomplished by CM [18]. Grubbs et al. recognised that CM can be selective when two partners showing different reactivity are used, e.g., reactive terminal olefin (type I) and an electron-deficient olefin (e.g., acrylates or acrylonitriles, type II or III). In these cases, full conversion and high yields can be achieved [18]. Although the problem with the CM reactions of olefins with electron-withdrawing groups, such as α,β-unsaturated ketones and esters, is partly solved [19–20], the conditions of the reactions need some improvement (lower catalyst loading, lower temperature). To the best of our knowledge, there is no such type of reaction performed at 0 °C. A lower temperature of the reaction is important in the synthesis of unstable and thermally-sensitive natural products.

**Figure 2:**
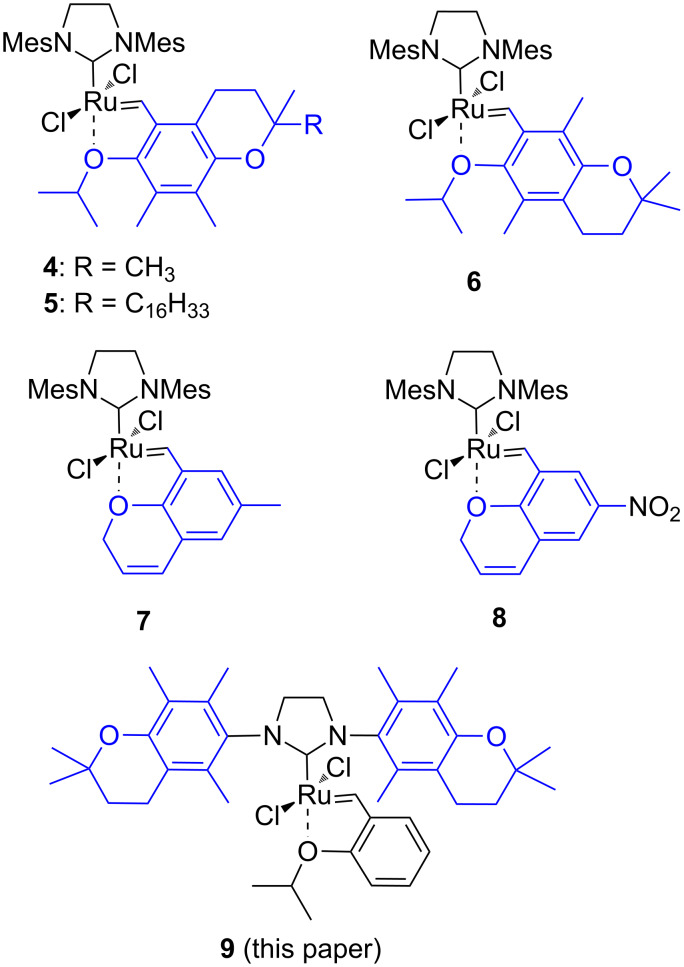
Selected ruthenium metathesis catalyst bearing chromanyl moieties.

We previously reported a few catalysts bearing the chromanyl moiety, derived from vitamin E [21–23]. In such a system as 2,2,5,7,8-pentamethyl-6-hydroxychromane (α-tocopherol model compound) specific stereoelectronic effects are observed [24–25], which might improve the activity of the catalyst bearing the above-mentioned moieties. The ruthenium complexes **4**–**6** (Figure 2) that we reported earlier appeared to be the so-called dormant catalysts. Their activity in RCM reactions was low at room temperature and higher at elevated temperature [21]. In catalyst **7** the chelating oxygen atom was provided by the rigid heterocyclic ring of the chromenylmethylidene moiety, whereas in the Hoveyda–Grubbs 2^nd^ generation catalyst, the complexing oxygen atom comes from the freely rotating isopropoxy substituent. This complex proved to be quite efficient and showed activity comparable to that of commercially available catalysts **1** and **2** [23]. The introduction of a nitro group into the 6-position of the chromene moiety in catalyst **8** led to a decrease in the stability of the complex [22].

The aforementioned modifications concerned the benzylidene moiety of the catalysts and affected the initiation rate of the metathesis reaction. Changes in the NHC ligand are also very important because this part of the catalyst participates all along the metathetic process. As a result, the catalyst may gain new properties, increased activity or stereoselectivity. Following this idea, we decided to synthesise a new catalyst bearing two chromanyl moieties symmetrically N,N’-disubstituted in the imidazolinium ring (**9**, Figure 2). According to Smith et al. [26] α-tocopherol and its amino analogue (α-tocopheramine) have comparable properties, coming from the same stereoelectronic effects mentioned above [24–25]. We expected that these effects may confer new properties of the NHC ligand.

## Results and Discussion

### Synthesis of the carbene precursor

**Scheme 1:**
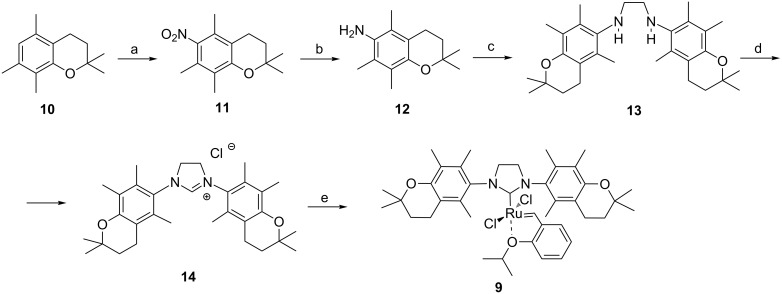
Synthesis of the new NHC precursor. Reagents and conditions: a) HNO_3_, CH_2_Cl_2_, 0 °C, 58%; b) HOCH_2_SOONa, Te, NaOH, dioxane, 50 °C, 50%; c) 2,3-dihydroxy-1,4-dioxane, EtOH, HCOOH, rt, then NaBH_3_CN, rt, 90%; d) NH_4_Cl, HC(OMe)_3_, reflux, 73%; e) I) *t*-AmOK, toluene, rt; II) Hoveyda–Grubbs 1^st^ generation, toluene, 65 °C, 68%.

The synthesis of an imidazolinium salt as a carbene precursor was started from 2,2,5,7,8-pentamethylchromane (**10**), which was prepared by the reaction between 2,3,5-trimethylphenol and 3-methylbut-2-enol [27]. Chromane **10** was nitrated with fuming nitric acid to give 6-nitrochromane **11** in 58% yield according to Mahdavian [28] (Scheme 1). Nitration using the Smith procedure [29] led to the expected nitrochromane **11**, however, formation of an admixture of 5a,6-dinitrochromane was observed. Reduction of the nitro group in **11** was slightly troublesome, probably due to steric hindrance. The tellurium-rongalit system was found to be the most efficient [30], and 6-chromanylamine **12** was obtained in 50% yield. Imidazolinium salt **14** was prepared according to the classical protocol. Chromanylamine **12** was subjected to reaction with 2,3-dihydroxy-1,4-dioxane (glyoxal equivalent) followed by reduction of the intermediate diimines by NaBH_3_CN to give ethylenediamine **13** in 90% yield. Usage of the more convenient sodium borohydride led to a prolonged reaction time (up to 20 h) and a lower reaction yield. Imidazolinium salt **14** was obtained by treatment of **13** with trimethyl orthoformate in 73% yield. It is worth noting that ethylenediamine **13** and imidazolinium salt **14** were sufficiently pure after precipitation, thus chromatographic purification was not necessary.

Some specific effects are observed in the NMR spectra of salt **14**. In the ^1^H NMR spectrum, the signals from protons of the imidazolinium ring have atypical multiplicity. Two neighbouring triplets are also present besides the expected singlet from the ethylene bridge between the two nitrogen atoms symmetrically substituted by two identical chromenyl moieties. Similarly, the C-2 protons give two singlets instead of one. We suspected that there is a hindered rotation on the *N*–C_(chromenyl)_ bond, so that two conformers are observed by NMR. However, the ^1^H NMR spectrum recorded at elevated temperatures (30 and 50 °C) looked the same. In the ^13^C NMR spectrum, signals from some of the carbon atoms are doubled. The HSQC correlation confirms that the doubled signals derive from one carbon atom. This fact also may suggest that two conformers are observed in the NMR spectra. This issue will be the subject of future detailed investigations.

### Synthesis of the catalyst

The new catalyst, **9**, was obtained from imidazolinium salt **14** by deprotonation with potassium *tert*-amylate followed by the tricyclohexylphosphine ligand exchange in Hoveyda–Grubbs 1^st^ generation catalyst to give the target catalyst **9**, which was purified on silica gel. The complex was stable in solid state at 0 °C for a few weeks (NMR test).

Attempts to obtain a monocrystal that would be suitable for X-ray analysis failed. The ^1^H NMR spectrum confirmed the structure of the catalyst. In the ^13^C NMR spectrum, some signals were broad and weak, e.g., signals of the quaternary aromatic carbon atoms and the primary carbon atoms of the methyl group attached to the aromatic ring of the chromanyl moieties. The HSQC correlation confirmed the structure of the catalyst and showed good correlation of the attached proton signals with the weak carbon peaks. When the ^13^C NMR spectrum is recorded at 50 °C, the spectrum becomes simpler but still many signals are almost invisible (at the noise level). The mass spectrum (HRMS) of **9** clearly evidences the molecular weight and the elemental composition of the new catalyst. The most likely unusual NMR spectra of **9** can result from specific stereoelectronic effects observed in the chromanyl system [24–25]. The 2p-type lone pair of electrons of the heterocyclic ring oxygen atom adopts an orientation almost perpendicular to the plane of the aromatic ring allowing for an electronic interaction with the *para*-substituent of the chromanyl system (–OH in 6-hydroxychroman and –NH_2_ in 6-aminochroman).

### Testing of the new catalyst

The catalyst proved very active in model cross-metathesis reactions, especially with olefins containing electron-withdrawing groups. For example the reaction of allylbenzene and ethyl acrylate at room temperature was almost quantitative. These results prompted us to test this reaction at a lower temperature (0 °C). Commercially available catalysts **1** and **2** proved inactive under these conditions, therefore we compared our catalyst with Grela catalyst **3** [17]. As is shown in Table 1 (entry 1), both catalysts, i.e., **3** and **9** proved very active. After reduction of the catalyst loading to 1 mol %, the yields of the ethyl acrylate reaction with allylbenzene catalysed by both complexes **3** and **9** were still very good (close to 80%, Table 1, entry 2). Encouraged by these results, we decided to test **9** in a CM reaction with other α,β-unsaturated compounds, e.g., methyl vinyl ketone (entry 3, Table 1) and acrylonitrile (entry 4, Table 1). The outcome of these reactions was also very promising for **9**. It should be added that in all cases a dimer of the electron-deficient olefin was not observed. The homodimerisation product of allylbenzene (entries 1 and 2, Table 1) was formed in less than 10% (in case of the reaction catalysed by **2** no dimeric products were observed), while the yield of the homodimer of hex-5-enyl acetate (entries 3 and 4, Table 1) was below 3%. Low conversion (especially that obtained with **1** and **2**) was related to substantial amounts of unreacted substrates.

**Table 1:** Comparative investigation of catalysts in CM reactions with electron-deficient olefins.

Entry	Alkene	Electron-deficient olefin	Product	Conditions^b^	Catalyst	Yield (*E*/*Z*)^c^

1	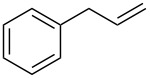	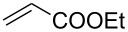	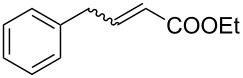	0 °C, 3 h, CH_2_Cl_2_ 2.5 mol % [Ru]	**1** **2** **3** **9**	11% (*E*/*Z* 100:1) 13% (*E*/*Z* 32:1) 87% (*E*/*Z* 29:1) 91% (*E*/*Z* 23:1)
2	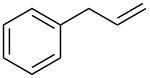	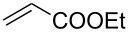	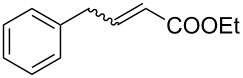	0 °C, 3 h, CH_2_Cl_2_ 1 mol % [Ru]	**3** **9**	77% 76%
3	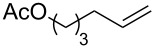	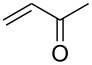	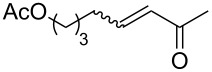	0 °C, 3 h, CH_2_Cl_2_ 1 mol % [Ru]	**1** **2** **3** **9**	0% 38% (only *E*) 98% (only *E*) 99% (only *E*)
4	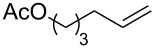	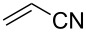	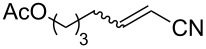	0 °C, 1 h, CH_2_Cl_2_ 1 mol % [Ru]	**1** **2** **3** **9**	0% 59% (*E*/*Z* 2.5:1) 75% (*E*/*Z* 3:1) 97% (*E*/*Z* 2.5:1)

^a^Electron-deficient olefin was used in excess (2 equiv). ^b^Concentration of alkene amounted 0.1 M. ^c^Determined by ^1^H NMR.

**Scheme 2:**
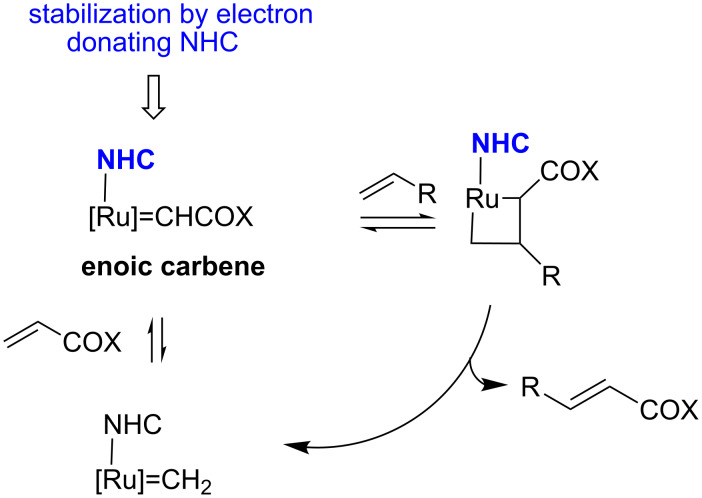
CM with electron-deficient olefin.

The high activity of Grela catalyst **3** was a result of faster initiation of the catalytic cycle arising from the electron-withdrawing effect of the nitro group. Consequently, lowered electron density at the oxygen atom in the isopropoxybenzylidene fragment weakens the coordination to the ruthenium atom, and finally, facilitates the initiating process. In the new catalyst **9**, which is modified in the NHC ligand, different effects are responsible for its activity. According to Grubbs [31], catalyst **1** is able to react with α,β-unsaturated carbonyl compounds to form an enoic carbene [Ru]=CHCOX, which is kinetically favourable. As a result, a stronger electron-donating ligand should stabilise the electron-deficient enoic carbene [31]. One can speculate that specific stereoelectronic effects occurring in the chromanyl system, known from the vitamin E chemistry, contribute to high activity of **9**. The *N*-(2,2,5,7,8-pentamethyl-6-chromanyl) substituents, bearing electron releasing methyl groups as well as interplaying dihydropyranyl oxygen and nitrogen atoms in the imidazolidine cycle, can stabilise the enoic carbene (Scheme 2).

The CM products of terminal olefins (entries 1–3, Table 2) were obtained in high and very high yields. Alkenes were converted almost quantitatively and the excess of (*Z*)-but-2-ene-1,4-diol diacetate was recovered. Some side products of self-metathesis (SM) of terminal alkenes were isolated. It should be added that two terminal olefins (entry 4, Table 2) gave also SM products besides the desired CM products. Furthermore, more dimeric products gave allyloxybenzene than hex-5-enyl acetate. It is worth noting that the CM reaction between styrene and (*Z*)-but-2-ene-1,4-diol diacetate (entry 1, Table 2) was highly efficient (97% yield) using catalyst **9**. Moreover, in the CM of allyloxybenzene and hex-5-enyl acetate, the yield with **9** was almost twice higher than that obtained for **1** or **2** (entry 4, Table 2). In the model RCM, the activity of catalyst **9** was slightly lower than that of commercial complexes, supposedly due to steric reasons (Table 3).

**Table 2:** Comparative investigation of the catalysts’ performance in CM reactions.

Entry	Substrates		Product	Catalyst	Yield (*E*/*Z*)^a^

1^b^	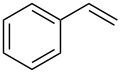	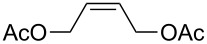	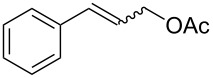	**1** **2** **9**	81% (*E*/*Z* 58:1) 80% (*E*/*Z* 43:1) 97% (*E*/*Z* 42:1)
2^b^	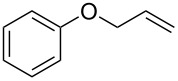	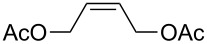	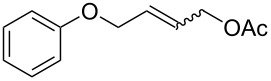	**1** **2** **9**	76% (*E/Z* 12:1) 75% (*E*/*Z* 12:1) 73% (*E*/*Z* 18:1)
3^b^	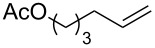	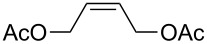	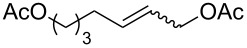	**1** **2** **9**	82% (*E*/*Z* 5:1) 86% (*E*/*Z* 5:1) 75% (*E*/*Z* 6:1)
4^c^	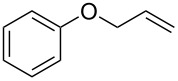	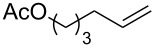	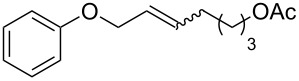	**1** **2** **9**	47% (*E*/*Z* 7:1) 53% (*E*/*Z* 5:1) 83% (*E*/*Z* 9:1)

^a^*E*/*Z* ratio determined by ^1^H NMR, isolated yield. ^b^Reaction conditions: 20 °C, 3 h, CH_2_Cl_2_, 0.1 M (terminal alkene), 2.5 mol % [Ru], *(Z)*-but-2-ene-1,4-diol diacetate (2 equiv). ^c^Reaction conditions: 20 °C, 3 h, CH_2_Cl_2_, 0.1 M (both alkenes), 2.5 mol % [Ru].

**Table 3:** Comparative investigation of catalysts in RCM.

Entry	Substrate	Product	Conditions	Catalyst	Conversion^a^

1	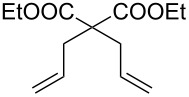	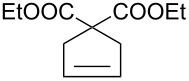	20 °C, CH_2_Cl_2_, 0.1 M, 1 h, 1 mol % [Ru]	**1** **2** **9**	95% 99% 70%
2	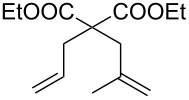	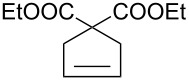	20 °C, CH_2_Cl_2_, 0.1 M, 1 h, 1 mol % [Ru]	**1** **2** **9**	83% 85% 65%
3	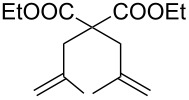	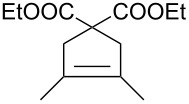	80 °C, toluene, 0.06 M, 16 h, 5 mol % [Ru]	**1** **2** **9**	38% 15% 22%

^a^Determined by ^1^H NMR.

**Scheme 3:**
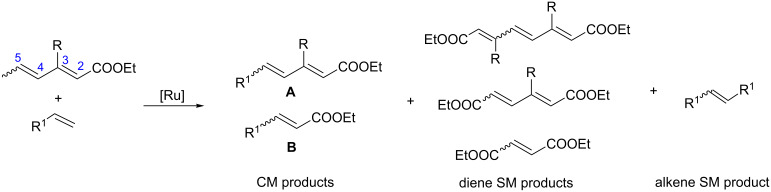
Possible products of metathesis reaction between diene and alkene.

The potency of the new catalyst **9** was tested not only in standard, model metathesis reactions but was also examined in more demanding systems, such as conjugated dienes and polyenes (Table 4). The CM reaction between alkene and diene (or polyene) often suffers from low regio- and stereoselectivity control. The CM reaction may be accompanied by various self-metathesis processes. Additionally, due to the competitive cleavage of both double bonds of the diene substrate, two different products may be formed in the CM reaction between alkene and diene (Scheme 3). A further complication is a *Z*/*E* isomer mixture formation.

**Table 4:** CM reactions between dienes and alkenes in the presence of various 2^nd^-generation catalysts^a^.

Entry	Diene	Alkene	Products	[Ru]	Yield^b^ (*E*/*Z*) product **A**	Yield (*E*/*Z*) product **B**

1^c^	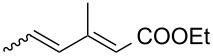	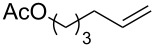	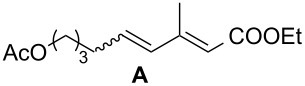	**1** **2** **9**	89% (only *E*) 72% (only *E*) 70% (only *E*)	– – –
2^d^	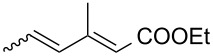	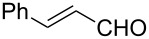	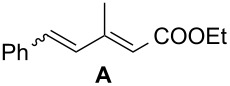	**1** **2** **9**	95% (only *E*) 92% (only *E*) 94% (only *E*)	– – –
3^d^	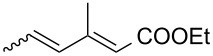	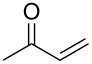	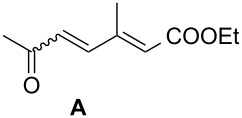	**1** **2** **9**	42% (only *E*) 40% (only *E*) 51% (only *E*)	– – –
4^c,e^	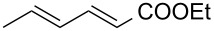	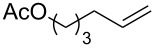	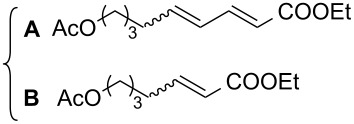	**1** **2** **9**	40% (*E*/*Z* 12:1) 20% (*E*/*Z* 11:1) 17% (*E*/*Z* 8:1)	10% (only *E*) 22% (*E*/*Z* 8:1) 49% (*E*/*Z* 18:1)

^a^Reaction conditions: diene (3 equiv, 0.36 M), alkene (1 equiv, 0.12 M), and 10 mol % of catalyst in DCM or toluene at 45 °C for 16 h. ^b^Isolated yield. Yields were calculated in relation to alkene. ^c^*E*/*Z* ratio determined by GC/MS. ^d^*E*/*Z* ratio determined by ^1^H NMR. ^e^Isolated as inseparable mixture of **A** and **B**. Yield calculated from ^1^H NMR.

The reactions of ethyl sorbate and its 3-methyl substituted analogue (ethyl (2*E*,4*Z*/*E*)-3-methylhexa-2,4-dienoate) with various alkenes were chosen to examine the activity and selectivity profile of catalyst **9** (Table 4). The results clearly indicate that carbene **9** can promote the CM reactions of dienes with different olefins as efficiently as commercial Grubbs 2^nd^ generation and Hoveyda–Grubbs 2^nd^ generation complexes **1** and **2**. Complex **9** catalysed the reactions of ethyl 3-methylhexa-2,4-dienoate in a completely selective manner taking into account product regio- and stereoselectivity. In all reactions the *E-*isomer of compound **A** was formed as a main product (Table 4, entries 1–3). Homodimerisation products of diene and alkene were obtained in very small amounts (<2% and <4% for entry 1 and 2, respectively, Table 4). The SM product of methyl vinyl ketone was not observed (entry 3, Table 4). Additionally, isomerised diene (ethyl (2*E*,4*E*)-3-methylhexa-2,4-dienoate, used in excess) was isolated from the reaction mixture. When ethyl sorbate was used, the formation of two CM products, **A** and **B**, was observed due to unselective metathetic scission of the C2–C3 and C4–C5 double bonds (Table 4, entry 4) [32]. The low product yield was caused not only by the unselective attack of catalyst on diene substrate but also by competitive SM reactions of both substrates.

**Figure 3:**
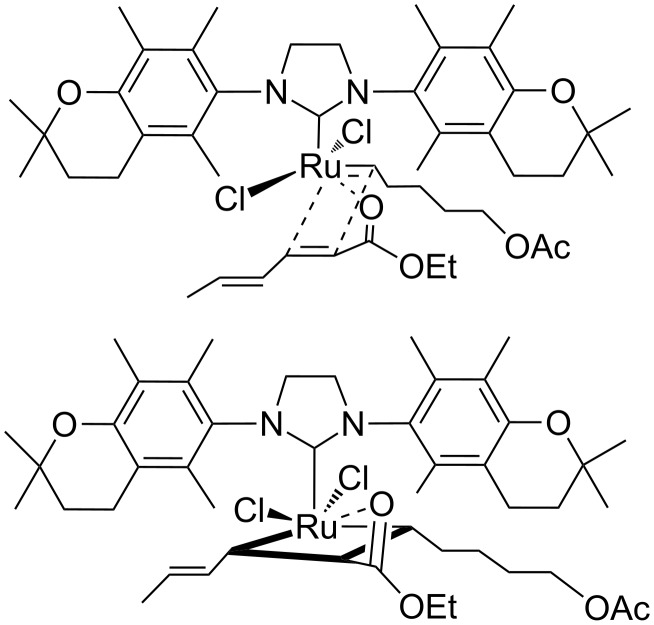
π-Complex and rutenacyclobutane intermediate with a five-membered ring chelate.

Interestingly, the new ruthenium complex showed a different regioselectivity compared to catalysts **1** and **2**. The metathetic scission of the less reactive C2–C3 double bond prevailed with complex **9** (Table 4, entry 4). This result could be explained by the higher affinity of the new catalyst toward electron-deficient olefins. It may also be assumed that the presence of the chromane system changes the electronic properties of the complex compared to other catalysts. It may favour chelation of the ruthenium centre of the catalyst by the carbonyl ester oxygen from the diene substrate. It seems that the oxygen–ruthenium bond is rather labile. The oxygen coordination does not decrease the activity of the catalyst but rather stabilises the π-complex and rutenacyclobutane intermediate (Figure 3), which promotes the metathesis reaction on the C2–C3 double bond. The formation of four- and five-membered chelates has been postulated previously [33–34].

**Scheme 4:**
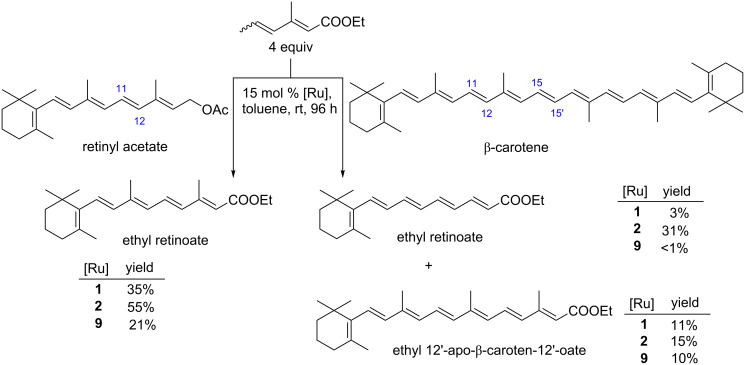
CM reaction of β-carotene and retinyl acetate with ethyl (2*E*,4*Z*/*E*)-3-methylhexa-2,4-dienoate. Reaction conditions: diene (4 equiv, 0.325 M), alkene (1 eqiuv, 0.08 M). Yield was obtained by quantative HPLC analysis. Yields were calculated in relation to polyene (retinyl acetate or β-carotene). In case of the β-carotene reaction, yields were divided by two due to the symmetry of the β-carotene molecule.

The CM reactions of polyenes, β-carotene and retinyl acetate with ethyl 3-methylhexa-2,4-dienoate in the presence of catalyst **9** were also studied (Scheme 4). A previous investigation of the CM reaction of β-carotene promoted by commercial Grubbs and Hoveyda–Grubbs 2^nd^ generation catalysts (**1** and **2**) proved that the two double bonds, C11–C12 and C15–C15’ of the β-carotene molecule were reactive in CM [35]. In the case of metathetic fragmentation of β-carotene, the use of **9** instead of **1** or **2** improved the regioselectivity. The product of the central C15–C15’ double bond scission was formed preferably. The activity of **9** was comparable to that of **1**, albeit lower than **2** (Scheme 4). Regio- and stereoselectivity (>95% *E*-isomer) of retinyl acetate CM catalysed by **9** appeared to be the same as in analogous reactions that were carried out in the presence of catalysts **1** and **2** [36] (Scheme 4). However, the product was obtained in a slightly lower yield.

## Conclusion

In summary, an efficient synthesis of new olefin metathesis catalyst **9** is reported. The catalyst contains a NHC ligand generated from the imidazolinium salt **14** bearing two symmetrically substituted 6-chromanyl moieties. The catalyst **9** was tested in model CM and RCM reactions and showed an activity comparable or superior to that of the commercial Grubbs and Hoveyda–Grubbs 2^nd^ generation complexes. The new complex was also active in metathesis of more demanding systems such as conjugated dienes and polyenes. It proved reactive toward electron-deficient olefins even at lowered temperature (0 °C).

## Experimental

### General

**Figure 4:**
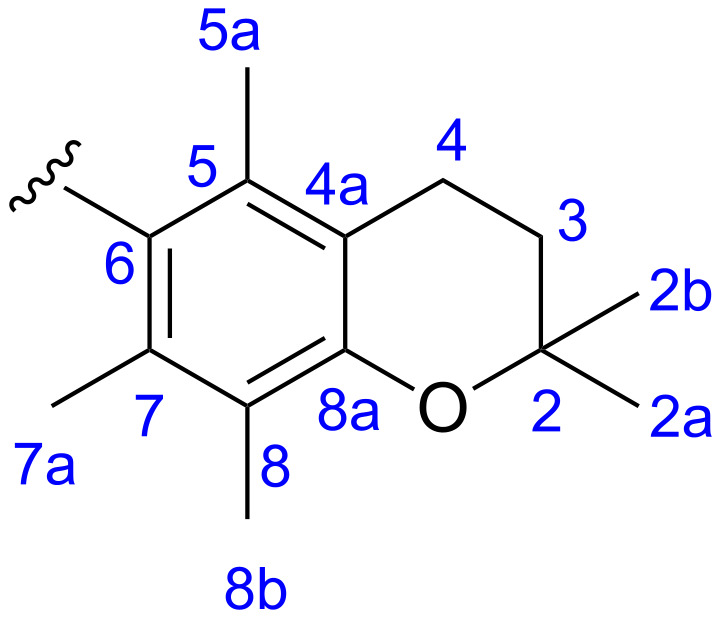
Numbering of carbon atoms in the chromanyl moiety.

All manipulations of organometallic compounds were performed using standard Schlenk techniques under an atmosphere of dry argon. CH_2_Cl_2_ was dried by distillation over CaH_2_, toluene over Na. 1,4-Dioxane, ethanol (96%) and trimethyl orthoformate were used as received. Melting points were determined on a Kofler apparatus of the Boetius type and were uncorrected. ^1^H and ^13^C NMR spectra were recorded on a Bruker Avance II spectrometer (400 and 100 MHz, respectively). Spectra are referenced relative to the chemical shift (δ) of TMS. Mass spectra were obtained with Micromass LCT TOF and AutoSpec Premier (Waters) spectrometers. IR spectra were recorded on a Nicolet series II Magna-IR 550 FTIR spectrometer. Flash chromatography (FC) was performed on silica gel 230–400 mesh. Catalysts: **1**, **2**, **3** and the Hoveyda–Grubbs 1^st^ generation complex were purchased from Apeiron Synthesis. 2,2,5,7,8-Pentamethylchromane (**10**) was prepared from 2,3,5-trimethylphenol analogously to the procedure described for 2,2,5,8-tetramethyl-6-chromanol by Dean [27]. 2,3-Dihydroxy-1,4-dioxane was obtain according to Venuti [37]. Substrates for testing catalysts in RCM reactions were prepared by allylation of commercial diethyl malonate with allyl bromide and/or 3-chloro-2-methylpropene according to Hensle [38]. Their purity was estimated by ^1^H NMR spectroscopy and found to be at least 95%. Other chemicals are commercially available and used as received. The numbering of carbon atoms in the chromanyl moiety was used as shown in Figure 4.

### 6-Nitro-2,2,5,7,8-pentamethylchromane (**11**)

To the solution of 2,2,5,7,8-pentamethylchromane (**10**) (2.5 g, 0.012 mol) in dry CH_2_Cl_2_ (150 mL) cooled to 0 °C fuming nitric acid (1.5 mL, 3 equiv, 0.036 mol) was added in one portion. The reaction was carried out at 0 °C for 1.5 h. Next the reaction mixture was washed with saturated NaHCO_3_, dried over Na_2_SO_4_ and evaporated. The crude product was purified by column chromatography (hexane/ethyl acetate 20:1) to give a yellow solid (1.75 g, 58%). Mp 120–123 °C; IR (KBr) ν: 2977, 2934, 1522, 1368, 1112 cm^−1^; ^1^H NMR (400 MHz, CDCl_3_) δ 2.64 (t, *J* = 6.8 Hz, 2H, H-4), 2.15 and 2.12 (2s, 9H, H-5a, 7a, 8b), 1.83 (t, *J* = 6.8 Hz, 2H, H-3), 1.33 (s, 6H H-2a, 2b) ppm; ^13^C NMR (100 MHz, CDCl_3_) δ 152.6 (C-8a), 146.2 (C-6), 126.1 (C-8), 125.0 (C-5), 123.7 (C-7), 117.4 (C-4a), 74.0 (C-2), 32.4 (C-3), 26.7 (C-2a and 2b), 20.8 (C-4), 14.5, 13.7, 11.8 (C-5a, 7a and 8b) ppm. HRMS (ESI): [M + Na]^+^ calcd for C_14_H_19_NNaO_3_: 272.1257, found: 272.1252. Literature mp 125–126 °C; IR, ^1^H NMR and ^13^C NMR data are compatible with the data in [39].

### 2,2,5,7,8-Pentamethylchromanyl-6-amine (**12**)

To the solution of rongalite (sodium hydroxymethylsulfinate dihydrate) (4.62 g, 5 equiv, 30 mmol) in a solution of 1 M NaOH (150 mL) tellurium powder (154 mg, 0.2 equiv, 1.2 mmol) was added. Then the solution of 6-nitro-2,2,5,7,8-pentamethylchromane (**11**, 1.5 g, 6 mmol) in dioxane (20 mL) was added to the reaction mixture. The reaction was carried out at 50 °C for 8 h. The reaction mixture was filtered through a pad of Celite and the filtrate was extracted with methylene chloride (3 × 20 mL). The organic layers were combined and washed with brine and water, dried over Na_2_SO_4_ and evaporated. The crude product was purified by column chromatography (hexane/ethyl acetate 8:1) to give a white solid (660 mg, 50%). Mp 38–40 °C; IR (KBr) ν: 3455, 3364, 2975, 2921, 1625, 1447, 1420, 1269 cm^−1^; ^1^H NMR (400 MHz, CDCl_3_) δ 3.31 (s, 2H NH_2_), 2.67 (t, *J =* 6.9 Hz, 2H, H-4), 2.15, 2.12 and 2.08 (3s, 9H, H-5a, 7a, 8b), 1.80 (t, *J* = 6.9 Hz, 2H, H-3), 1.30 (s, 6H, H-2a, 2b) ppm; ^13^C NMR (100 MHz, CDCl_3_) δ 144.9 (C-8a), 134.9 (C-6), 122.2 (C-8), 120.4 (C-7), 117.6 (C-5), 116.9 (C-4a), 72.2 (C-2), 33.3 (C-3), 26.7 (C-2a and 2b), 21.4 (C-4), 13.5, 12.5, 11.9 (C-5a, 7a, 8b) ppm. HRMS (ESI): [M + H]^+^ calcd for C_14_H_22_NO: 220.1696, found: 220.1691. Literature mp 51.5–53.5 °C; IR, ^1^H NMR and ^13^C NMR data are compatible with the data in [39].

### *N*,*N*-Bis(2,2,5,7,8-pentamethylchroman-6-yl)ethane-1,2-diamine (**13**)

To a solution of 2,2,5,7,8-pentamethylchromanyl-6-amine (**12**, 500 mg, 2.3 mmol) in ethanol (96%, 20 mL), 2,3-dihydroxy-1,4-dioxane (138 mg, 1.15 mmol) and two drops of formic acid were added in a manner similar to a that described in [40]. The reaction mixture was stirred for 24 h. A yellow precipitate appeared. Then sodium cyanoborohydride (217 mg, 3 equiv, 3.45 mmol) was added and the reaction mixture was stirred for 1.5 h. The reaction mixture was quenched with water (20 mL) and extracted with dichloromethane (3 × 20 mL). The organic layers were washed with brine and water, dried over Na_2_SO_4_ and evaporated to dryness. After crystallization from ethanol a white solid was obtained (480 mg, 90%). Mp 182–183 °C; IR (KBr) ν: 3369, 2968, 2925, 2779, 1450, 1420, 1265 cm^−1^; ^1^H NMR (400 MHz, CDCl_3_) δ 3.04 (s, 4H, -NC*H*_2_C*H*_2_N-), 2.64 (t, *J* = 6.8 Hz, 4H, H-4), 2.26, 2.22 and 2.13 (3s, 18H, H-5a, 7a, 8b), 1.81 (t, *J* = 6.8 Hz, 4H, H-3), 1.31 (s, 12H, H-2a, 2b) ppm; ^13^C NMR (100 MHz, CDCl_3_) δ 147.9 (C-8a), 137.6 (C-6), 129.1 (C-8), 127.1 (C-7), 122.6 (C-5), 117.2 (C-4a), 72.6 (C-2), 50.4 (-NH-*C*H_2_-*C*H_2_-NH-), 33.1 (C-3), 26.8 (C-2a and 2b), 21.4 (C-4), 14.4, 13.5, 12.1 (C-5a, 7a, 8b) ppm. HRMS (ESI): [M + H]^+^ calcd for C_30_H_45_N_2_O_2_: 465.3476, found: 465.3465.

### 1,3-Bis(2,2,5,7,8-pentamethylchroman-6-yl)-4,5-dihydro-1*H*-imidazol-3-ium chloride (**14**)

To a solution of ethylenediamine **13** (200 mg, 0.43 mmol) in trimethyl orthoformate (5 mL), ammonium chloride (35 mg, 0.65 mmol) was added in a similar manner as described in [40]. The reaction mixture was refluxed for 2 h. Solids were filtered off and the filtrate was evaporated to dryness. The resulting oil was stirred overnight with diethyl ether causing precipitation of a white solid (160 mg, 73% yield). Mp 246–247 °C; IR (KBr) ν: 3397, 2974, 2929, 1626, 1456, 1411, 1269 cm^−1^; ^1^H NMR (400 MHz, CDCl_3_) δ 8.56 and 8.52 (2s, 1H, N-CH=N), 4.75–4.50 (m, 4H, -NC*H*_2_C*H*_2_N-), 2.64 (t, *J* = 6.8 Hz, 4H, H-4), 2.30, 2.28 and 2.12 (18H, 3s, H-5a, 7a, 8b), 1.82 (t, *J* = 6.8 Hz, 4H, H-3), 1.32 (s, 12H, H-2a, 2b) ppm; ^13^C NMR (100 MHz, CDCl_3_) δ 159.3, 159.2 (-N=*C*H-N-), 153.2 (C-8a), 131.8, 131.6 (C-6), 131.3, 131.0 (C-8), 124.4, 124.3 (C-7), 124.2 (C-5), 118.3, 118.2 (C-4a), 74.0 (C-2), 53.0 (-NH-*C*H_2_-*C*H_2_-NH-), 32.2 (C-3), 26.9, 26.8, 26.5, 26.47 (C-2a and 2b), 21.0 (C-4), 15.1, 15.0, 14.4, 14.3, 12.0 (C-5a, 7a, 8b) ppm; HRMS (ESI): [M − Cl]^+^ calcd for C_31_H_43_N_2_O_2_: 475.3319, found: 475.3308. Some signals in ^13^C NMR spectrum are doubled (see text “Synthesis of the carbene precursor”).

### 1,3-Bis[(2,2,5,7,8-pentamethylchroman-6-yl)-2-imidazolidinylidene]dichloro(*o*-isopropoxyphenylmethylene)ruthenium (**9**)

In a Schlenk flask imidazolinium salt **14** (80 mg, 0.16 mmol) was dried under vacuum at 80 °C for 1 h. The flask was then cooled to rt and dry toluene (3 mL) was added under argon atmosphere. To the resulting suspension potassium *tert*-amylate (1.7 M in toluene, 94 µL, 0.16 mmol) was added and the reaction mixture was stirred for 20 min at rt. Then a solution of Hoveyda–Grubbs 1^st^ generation catalyst (96 mg, 0.16 mmol) in toluene (3 mL) was added and the reaction was carried out at 65 °C for 2 h. The reaction mixture was purified by column chromatography (hexane/ethyl acetate 10:1) to give a green solid (86 mg, 68% yield) Mp 162–164 °C; IR (KBr) ν: 2973, 2925, 2854, 1475, 1457, 1274 cm^−1^; ^1^H NMR (400 MHz, CDCl_3_) δ 16.74 (s, 1H, Ru=C*H*-), 7.47, 6.94, 6.84 and 6.78 (4m, 4H, -CH_Ar_), 4.89 (sept., *J =* 6.1 Hz, 1H, -C*H*_iPr_), 4.16 (s, 4H, -N-C*H*_2_C*H*_2_-N-), 2.69 (m, 4H, H-4), 2.42, 2.29 and 2.20 (3s (2 br s and 1s), 18H, 3s H-5a, 7a, 8b), 1.88 (*t, J* = 6.5 Hz, 4H, H-3), 1.44 (s, 12H, H-2a, 2b), 1.28 (d, *J* = 6.0 Hz, 6H, CH_3 iPr_) ppm; ^13^C NMR (100 MHz, CDCl_3_) δ 297.5 (*C*H=Ru), 211.7 (C_NHC_), 152.1 (^IV^C_Ar_), 145.4 (^IV^C_Ar_), 135.0 (^IV^C_Ar weak_), 133.5 (^IV^C_Ar weak_), 132.2 (^IV^C_Ar weak_), 129.1 (^III^CH_Ar_), 129.0 (^IV^C_weak_), 123.3 (^IV^C_Ar weak_), 122.9 (^III^CH_Ar_), 122.4 (^III^CH_Ar_), 117.1 (^IV^C_Ar weak_), 112.8 (^III^CH_Ar_), 74.7 (CH_IPr_), 73.3 (C_chroman_-2), 53.1 and 51.8 (N-(*C*H_2_)_2_-N), 33.1 (C_chroman_-3), 27.9 (C_chroman_-2a and 2b), 25.7 (CH_3 IPr_), 21.2 (C_chroman_-4), 18.3 (CH_3 chroman weak_), 15.2 (CH_3 chroman weak_), 12.0 (CH_3 chroman_) ppm; EIMS *m*/*z*: 796 (10), 794 (10), 610 (14), 572 (7), 473 (41), 472 (100), 416 (31), 243 (7), 181 (8), 108 (12), 69 (14), 44 (14%); HRMS (EI): [M]^+^ calcd for C_41_H_54_Cl_2_N_2_O_3_^102^Ru: 794.2549, found: 794.2571. Some signals in ^13^C NMR spectrum are weak (see text “Synthesis of the catalyst”).

## Supporting Information

File 1 Experimental procedures of the testing of the new catalyst and copies of ^1^H and ^13^C NMR spectra of new compounds.
