# Supplementary material for: New metathesis catalyst bearing chromanyl moieties at the N-heterocyclic carbene ligand
Source: Beilstein J Org Chem. 2015 Dec 30;11:2795–804. doi: 10.3762/bjoc.11.300 (PMC4734414; doi:10.3762/bjoc.11.300)
Supplement: File 1 — Experimental procedures of the testing of the new catalyst and copies of 1H and 13C NMR spectra of new compounds. [file Beilstein_J_Org_Chem-11-2795-s001.pdf]

Supporting Information

for

New metathesis catalyst bearing chromanyl moieties at the N-heterocyclic carbene ligand

Agnieszka Hryniewicka*, Szymon Suchodolski, Agnieszka Wojtkielewicz, Jacek W. Morzycki and Stanisław Witkowski*

Address: University of Białystok, Institute of Chemistry, Ciołkowskiego Street 1K, 15-245 Białystok; Poland

Email: Agnieszka Hryniewicka - aga_h@uwb.edu.pl; Stanisław Witkowski - wit@uwb.edu.pl

*Corresponding author

Experimental procedures of the testing of the new catalyst and copies of ^{1}H and ^{13}C NMR spectra of new compounds

Table of contents

Testing of the new catalyst 9………………………………………………………………………………………………..… S3

Copies of NMR spectra of new compounds

^{1}H NMR spectrum of 6-nitro-2,2,5,7,8-pentamethylchromane (**11**)……………………………………………………………. S6

^{13}C NMR spectrum of 6-nitro-2,2,5,7,8-pentamethylchromane (**11**)……………………………………….……………..... S7

^{1}H NMR spectrum of 2,2,5,7,8-pentamethylchromanyl-6-amine (**12**)………………………………………………..........….. S8

^{13}C NMR spectrum of 2,2,5,7,8-pentamethylchromanyl-6-amine (**12**)………………………………………………………… S9

^{1}H NMR spectrum of *N*,*N*-*bis*(2,2,5,7,8-pentamethylchroman-6-yl)ethane-1,2-diamine (**13**)………………………………… S10

^{13}C NMR spectrum of *N*,*N*-*bis*(2,2,5,7,8-pentamethylchroman-6-yl)ethane-1,2-diamine (**13**)………………………………… S11

^{1}H NMR spectrum of 1,3-*bis*(2,2,5,7,8-pentamethylchroman-6-yl)-4,5-dihydro-1*H*-imidazol-3-ium chloride (**14**)……….….... S12

^{13}C NMR spectrum of 1,3-*bis*(2,2,5,7,8-pentamethylchroman-6-yl)-4,5-dihydro-1*H*-imidazol-3-ium chloride (**14**)………… S13

HSQC spectrum of 1,3-*bis*(2,2,5,7,8-pentamethylchroman-6-yl)-4,5-dihydro-1*H*-imidazol-3-ium chloride (**14**)……….……. S14

^{1}H NMR spectrum of the catalyst **9**……………………………………………………………………….……………...... S15

^{13}C NMR spectrum of the catalyst **9**…………………………………………………………………….………….…..... S16

HSQC spectrum of the catalyst **9**…………………………………………………………………………….……………. S17

HSQC spectrum of the catalyst **9** (limited range)………………………………………………………………….……… S18

^{1}H NMR spectrum of the catalyst **9** (T = 323 K; limited range)…………………………………………………….…….. S19

^{13}C NMR spectrum of the catalyst **9** (T = 323 K; limited range)…………………….…………………………………. S20

^{13}C DEPT spectrum of the catalyst **9** (T = 323 K; limited range)…………………….……………………............…. S21

References………………………………………………………………………………………….….. S22

Testing of the catalyst 9

General CM procedure for allylbenzene and ethyl acrylate (**Table 1**, **entries 1, 2**)

a) To a mixture of allylbenzene (0.2 mmol) and ethyl acrylate (0.4 mmol) in CH_2Cl_2 (1.5 mL) a solution of catalyst **1**, **2**, **3** or **9** (2.5 mol %) in CH_2Cl_2 (0.5 mL) was added. The resulting mixture was stirred at 0 °C under argon atmosphere and controlled by ^{1}H NMR after 1, 2 and 3 hours.

b) To a mixture of allylbenzene (0.2 mmol) and ethyl acrylate (0.4 mmol) in CH_2Cl_2 (1.5 mL) a solution of catalyst **3** or **9** (1 mol %) in CH_2Cl_2 (0.5 mL) was added. The resulting mixture was stirred at 0 °C under argon atmosphere and analysed by ^{1}H NMR after 3 hours.
The spectral data obtained for CM product are consistent with those given in the literature [1].

Yield was determined by comparison of integration of CM product proton signal (dd, 2H) at 3.53 ppm or (dt, 1H) at 5.83 ppm and SM product of allylbenzene ((*E*)-1,4-diphenylbut-2-ene) proton signal (m, 2H) at 5.67–5.71 ppm and/or substrate (allylbenzene, since acrylate was used in excess) proton signal (m, 1H) at 5.81–6.17 ppm. Amount of protons corresponding to these signals was taken into account in the calculations.

General CM procedure for hex-5-enyl acetate and methyl vinyl ketone (**Table 1**, **entry 3**)
To a mixture of the hex-5-enyl acetate (0.2 mmol) and methyl vinyl ketone (0.4 mmol) in CH_2Cl_2 (1.5 mL) a solution of catalyst **1**, **2**, **3** or **9** (1 mol %) in CH_2Cl_2 (0.5 mL) was added. The resulting mixture was stirred at 0 °C under argon atmosphere and controlled by ^{1}H NMR after 3 hours. The spectral data obtained for CM product are consistent with those given in the literature [2].
Yield was determined by comparison of integration of CM product proton signal (dt, 1H) at 6.07 ppm and SM product of hex-5-enyl acetate ((*E*)-dec-5-ene-1,10-diol diacetate) proton signal (m, 2H) at 5.40–5.42 ppm and/or substrate (hex-5-enyl acetate, since ketone was used in excess) proton signal (m, 1H) at 5.74–5.84 ppm. Amount of protons corresponding to these signals was taken into account in the calculations.

General CM procedure for hex-5-enyl acetate and acrylonitrile (**Table 1**, **entry 4**)
To a mixture of the hex-5-enyl acetate (0.2 mmol) and acrylonitrile (0.4 mmol) in CH_2Cl_2 (1.5 mL) a solution of catalyst **1**, **2**, **3** or **9** (1 mol %) in CH_2Cl_2 (0.5 mL) was added. The resulting mixture was stirred at 0 °C for 1 h under argon atmosphere and analysed by ^{1}H NMR. The spectral data obtained for CM product are consistent with those given in the literature [3].
Yield was determined by comparison of integration of CM product proton signal (d, 1H) at 5.35 ppm and SM product of hex-5-enyl acetate ((*E*)-dec-5-ene-1,10-diol diacetate) proton signal (m, 2H) at 5.40–5.42 ppm and/or substrate (hex-5-enyl acetate, since nitrile was used in excess) proton signal (m, 1H) at 5.74–5.84 ppm. Amount of protons corresponding to these signals was taken into account in the calculations.

General CM procedure for selected alkenes with (Z)-but-2-ene-1,4-diol diacetate (**Table 2**, **entries 1–3**)
To a mixture of the alkene (0.2 mmol) and *(Z)*-but-2-ene-1,4-diol diacetate (0.4 mmol) in CH_2Cl_2 (1.5 mL) a solution of catalyst **1**, **2** or **9** (2.5 mol %) in CH_2Cl_2 (0.5 mL) was added. The resulting mixture was stirred at 20 °C for 3 h under argon atmosphere. The crude product was

purified by FC (hexane – ethyl acetate v/v 50:1) and analysed by ^{1}H NMR. The spectral data obtained for CM products are consistent with those given in the literature [4].

General CM procedure for allyloxybenzene and hex-5-enyl acetate (**Table 2**, **entry 4**)
To a mixture of the alkenes (0.2 mmol, both) in CH_2Cl_2 (1.5 mL) a solution of catalyst **1**, **2** or **9** (2.5 mol %) in CH_2Cl_2 (0.5 mL) was added. The resulting mixture was stirred at 20 °C for 3 h under argon atmosphere. The crude product was purified by FC (hexane – ethyl acetate v/v 20:1) and analysed by ^{1}H NMR. The spectral data obtained for CM product are consistent with those given in the literature [4].

General RCM procedure for diethyl diallylmalonate (**Table 3**, **entry 1**)
To a solution of the alkene (0.2 mmol) in CH_2Cl_2 (1.5 mL) a solution of catalyst **1**, **2** or **9** (1 mol %) in CH_2Cl_2 (0.5 mL) was added. The resulting mixture was stirred at room temperature for 60 min under argon atmosphere. The crude product was analysed by ^{1}H NMR. The spectral data obtained for RCM product are consistent with those given in the literature [5].

General RCM procedure for diethyl allylmethallylmalonate (**Table 3**, **entry 2**)
To a solution of the alkene (0.2 mmol) in CH_2Cl_2 (1.5 mL) a solution of catalyst **1**, **2** or **9** (1 mol %) in CH_2Cl_2 (0.5 mL) was added. The resulting mixture was stirred at room temperature for 60 min under argon atmosphere. The crude product was analysed by ^{1}H NMR. The spectral data obtained for RCM product are consistent with those given in the literature [5].

General RCM procedure for diethyl dimethallylmalonate (**Table 3**, **entry 3**)
To a solution of alkene (0.2 mmol) in toluene (2 mL) a solution of catalyst **1**, **2** or **9** (5 mol %) in toluene (1 mL) was added. The resulting mixture was stirred at 80 °C for 16 h under argon atmosphere. The crude product was analysed by ^{1}H NMR. The spectral data obtained for RCM product are consistent with those given in the literature [5].

General CM procedure for diene and alkene (**Table 4**, **entries 1–4**)
To the solution of ethyl (2*E*,4*E*/Z)-3-methylhexa-2,4-dienoate or (2*E*,4*E*)-hexa-2,4-dienoate (0.36 mmol) and a catalyst **1**, **2** or **9** (10 mol %) in dry CH_2Cl_2 or toluene (0.5 mL) the solution of alkene (0.12 mmol) in dry DCM or toluene (0.5 mL) was added dropwise. The reaction mixture was stirred at 45 °C under argon atmosphere for 16 hours. Then the mixture was concentrated in vacuo and purified directly by silica gel chromatography using hexane/ethyl acetate as eluent. The diastereomeric ratio was determined by GC/MS or NMR analysis. The spectral data obtained for compounds: ethyl (2*E*,4*E*)-9-acetoxy-3-methylnona-2,4-dienoate [6] (entry 1, product A), ethyl (2*E*,4*E*)-3-methyl-5-phenylpenta-2,4-dienoate [7] (entry 2, product A), ethyl (2*E*,4*E*)-3-methyl-6-oxohepta-2,4-dienoate [8] (entry 3, product A), ethyl (2*E*,4*E*)-9-acetoxynona-2,4-dienoate [9] (entry 4, product A) and ethyl 7-acetoxyhept-2-enoate [10] (entry 4, product B) are consistent with those given in the literature.
In case of reaction between (2*E*,4*E*)-hexa-2,4-dienoate and hex-5-enyl acetate (entry 4) yield calculated from ^{1}H NMR spectrum of isolated mixture of inseparable products **A** and **B** by integration of product A proton signal (dd) at 7.26 ppm and product B proton signal (dt) at 6.95 ppm.

General CM procedure for polyene and diene (**Scheme 4**)

To the solution of a polyene (2.44×10^{-2} mmol) and a catalyst **1**, **2** or **9** (15 mol %) in dry toluene (0.3 mL) ethyl (2*E*,4*Z*/*E*)-3-methylhexa-2,4-dienoate (9.76×10^{-2} mmol) was added dropwise. The reaction mixture was stirred at room temperature for 96 hours under argon atmosphere. Then the mixture was analysed by HPLC and purified by semipreparative HPLC. The spectral data obtained for CM products: ethyl retinoate and ethyl 12'-apo-β-caroten-12'-oate are consistent with those given in the literature [11].

Copies of NMR spectra of new compounds

^{1}H NMR spectrum of 6-nitro-2,2,5,7,8-pentamethylchromane (**11**)

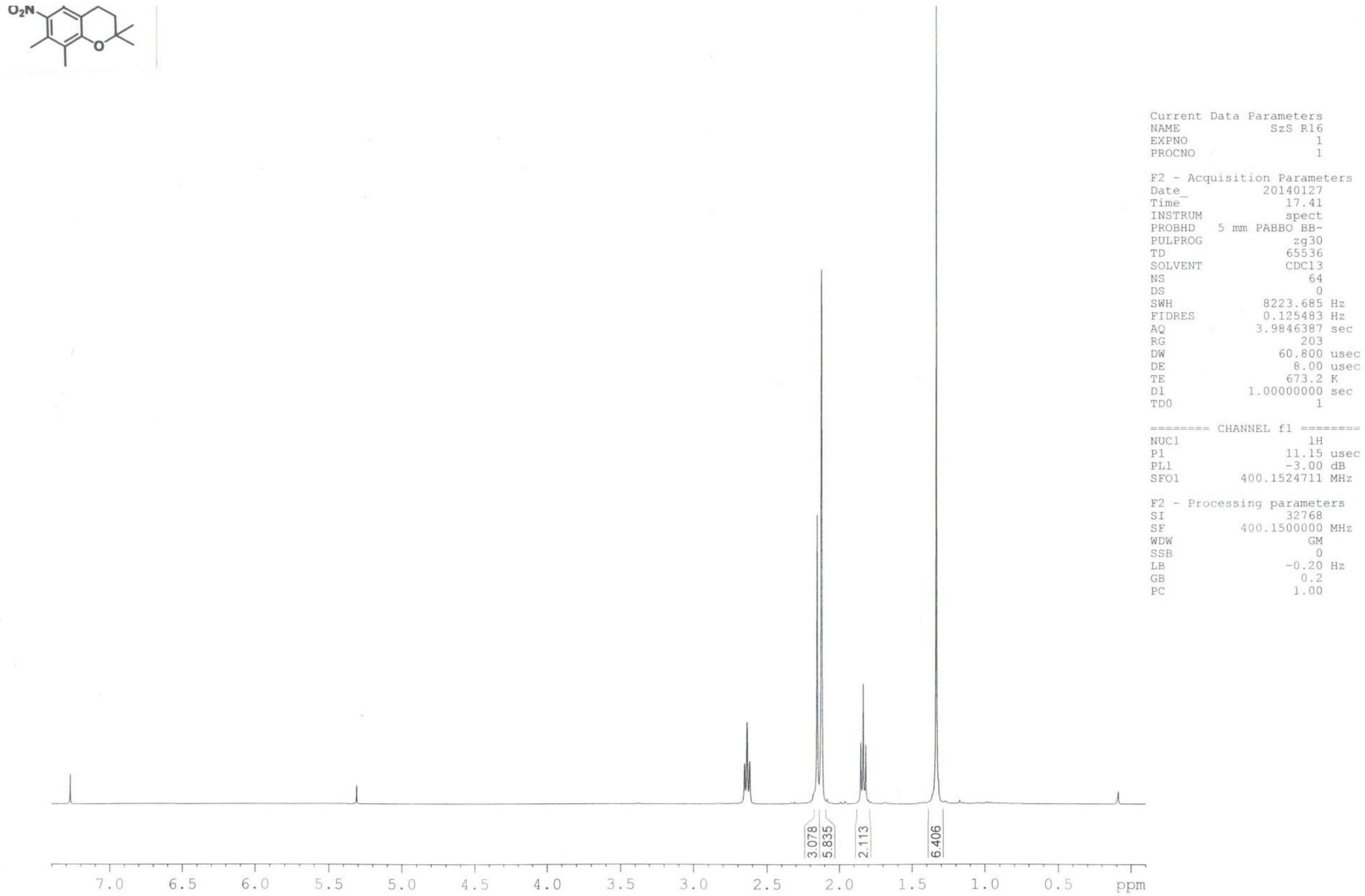

^{13}C NMR spectrum of 6-nitro-2,2,5,7,8-pentamethylchromane (**11**)

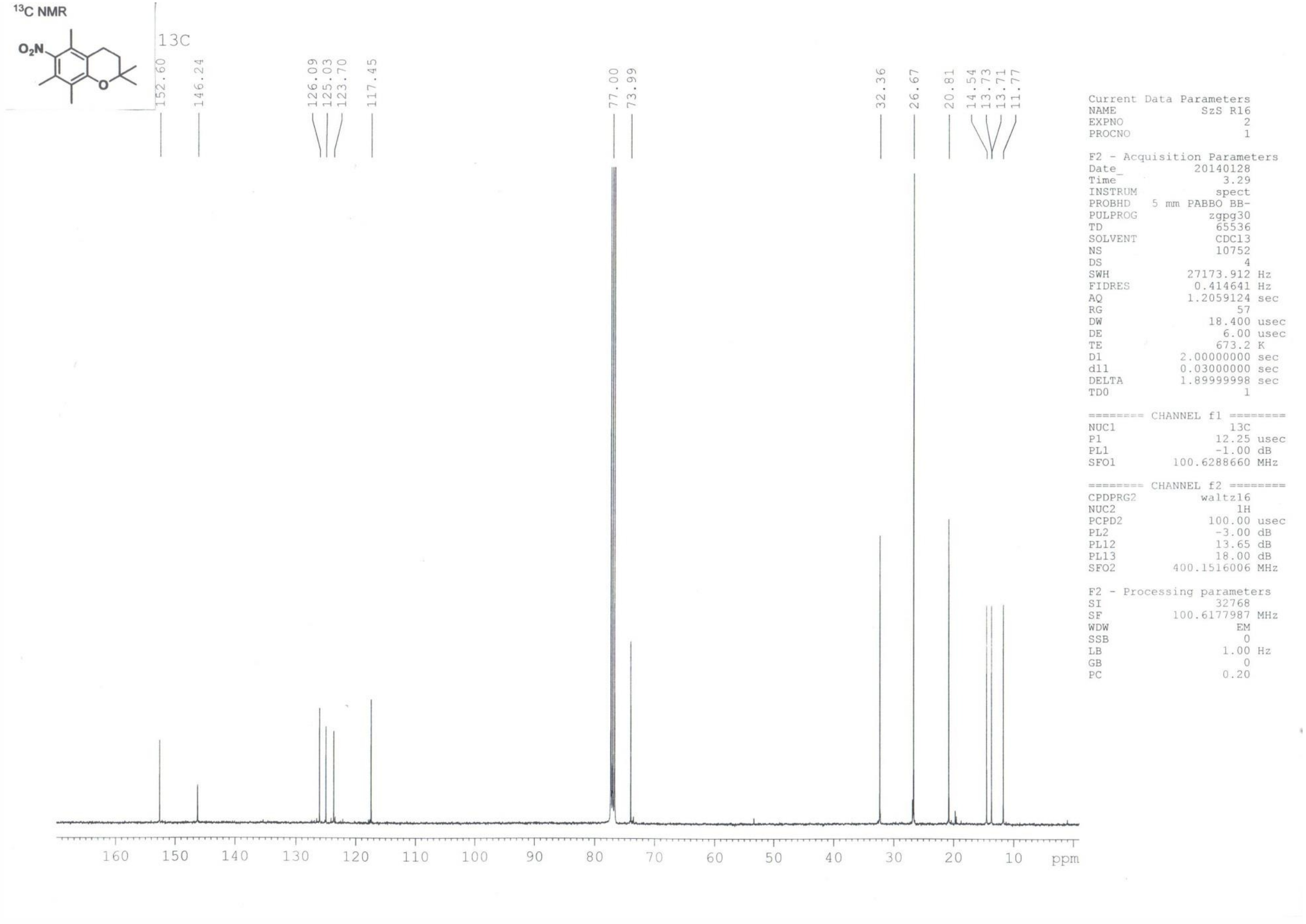

^{1}H NMR spectrum of 2,2,5,7,8-pentamethylchromanyl-6-amine (**12**)

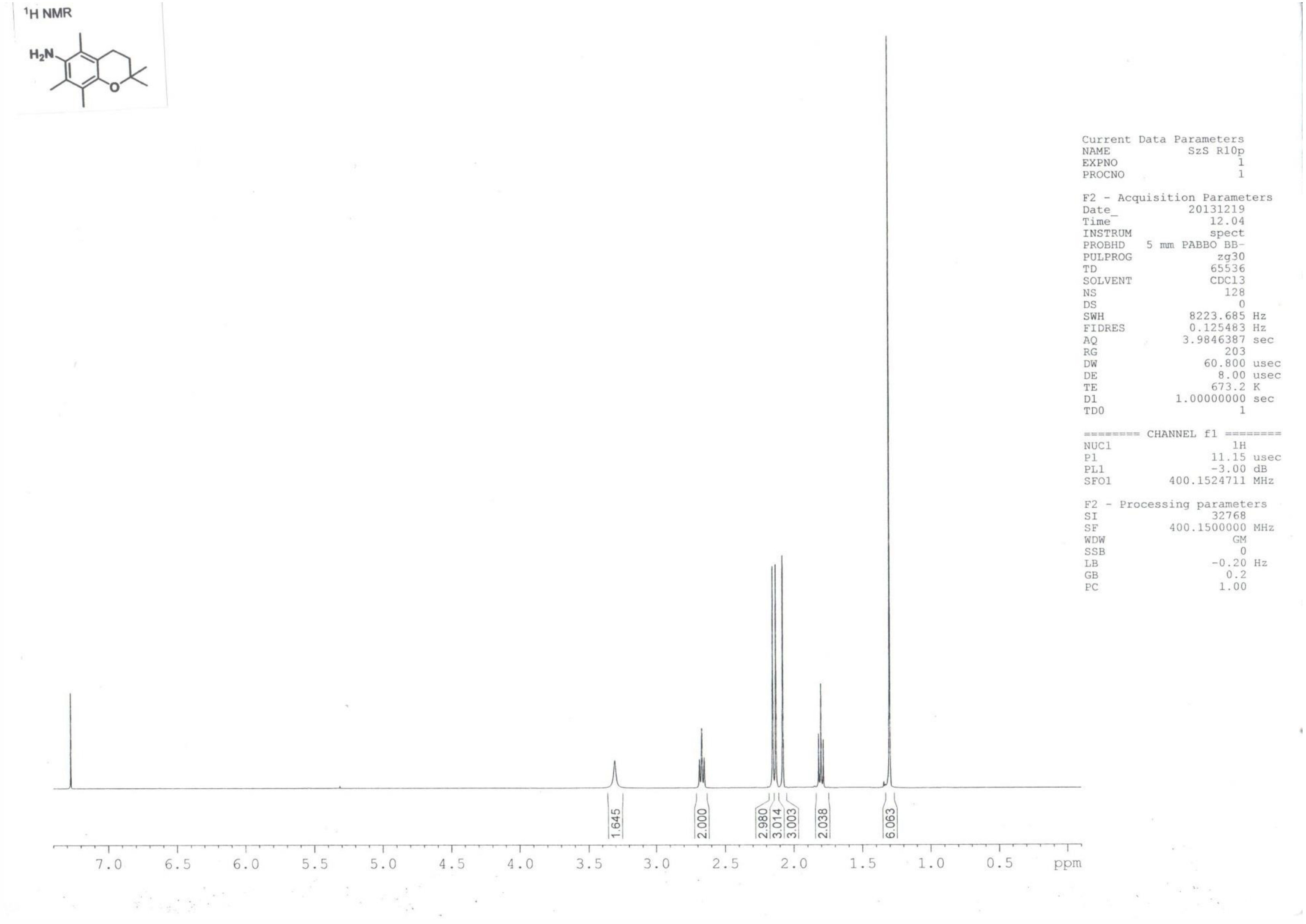

^{13}C NMR spectrum of 2,2,5,7,8-pentamethylchromanyl-6-amine (**12**)

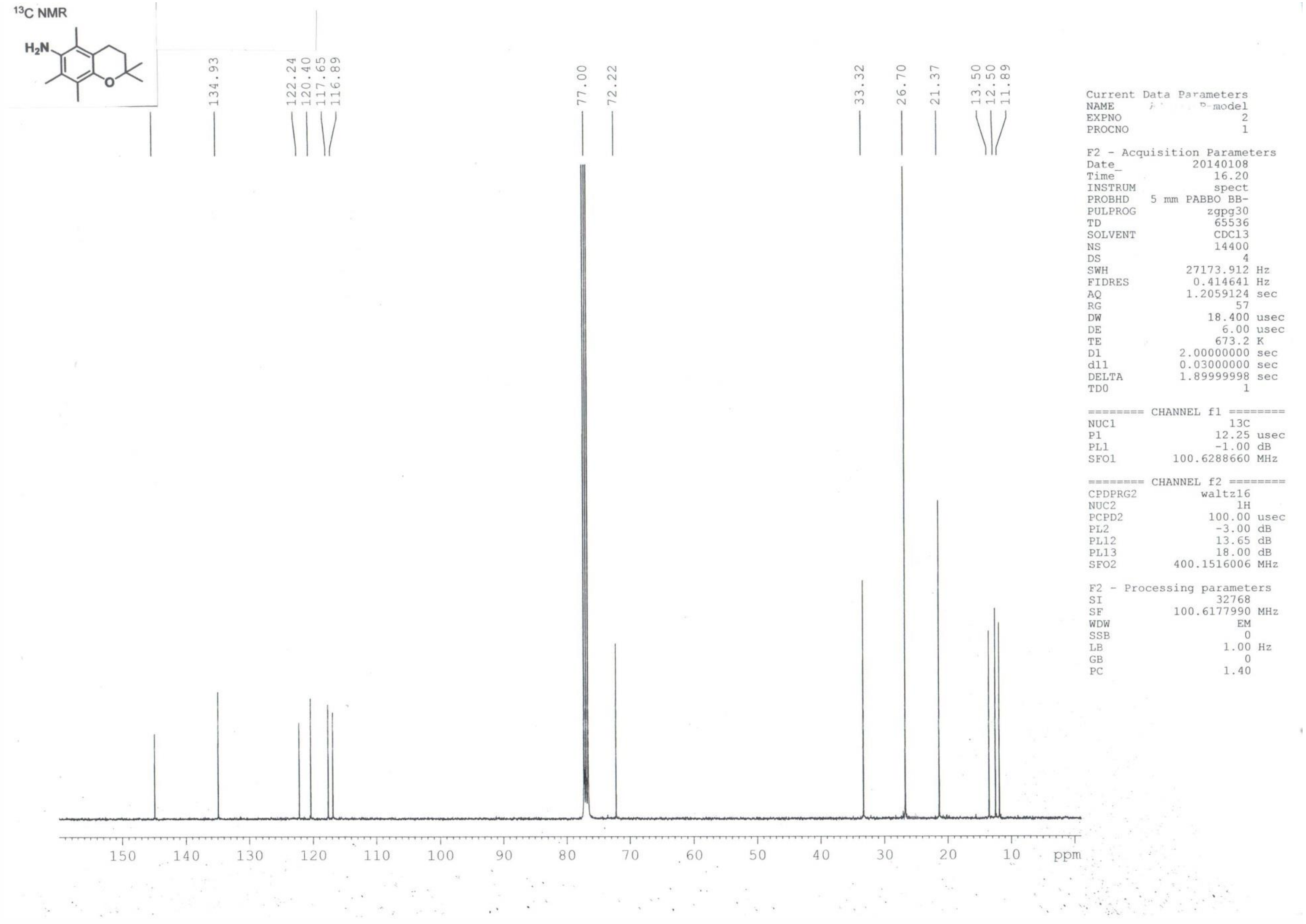

^{1}H NMR spectrum of *N*,*N*-*bis*(2,2,5,7,8-pentamethylchroman-6-yl)ethane-1,2-diamine (**13**)

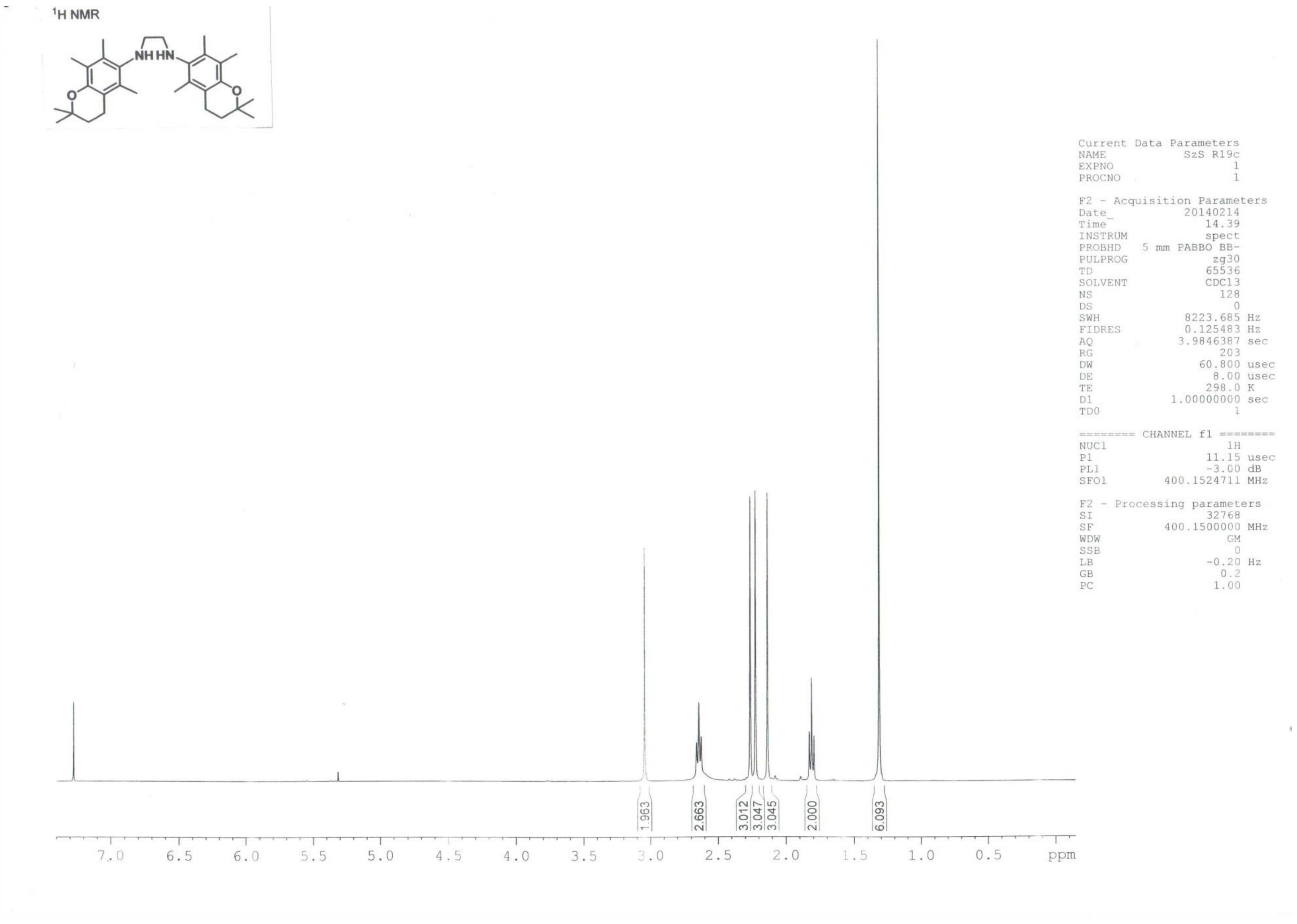

^{13}C NMR spectrum of *N*,*N*-*bis*(2,2,5,7,8-pentamethylchroman-6-yl)ethane-1,2-diamine (**13**)

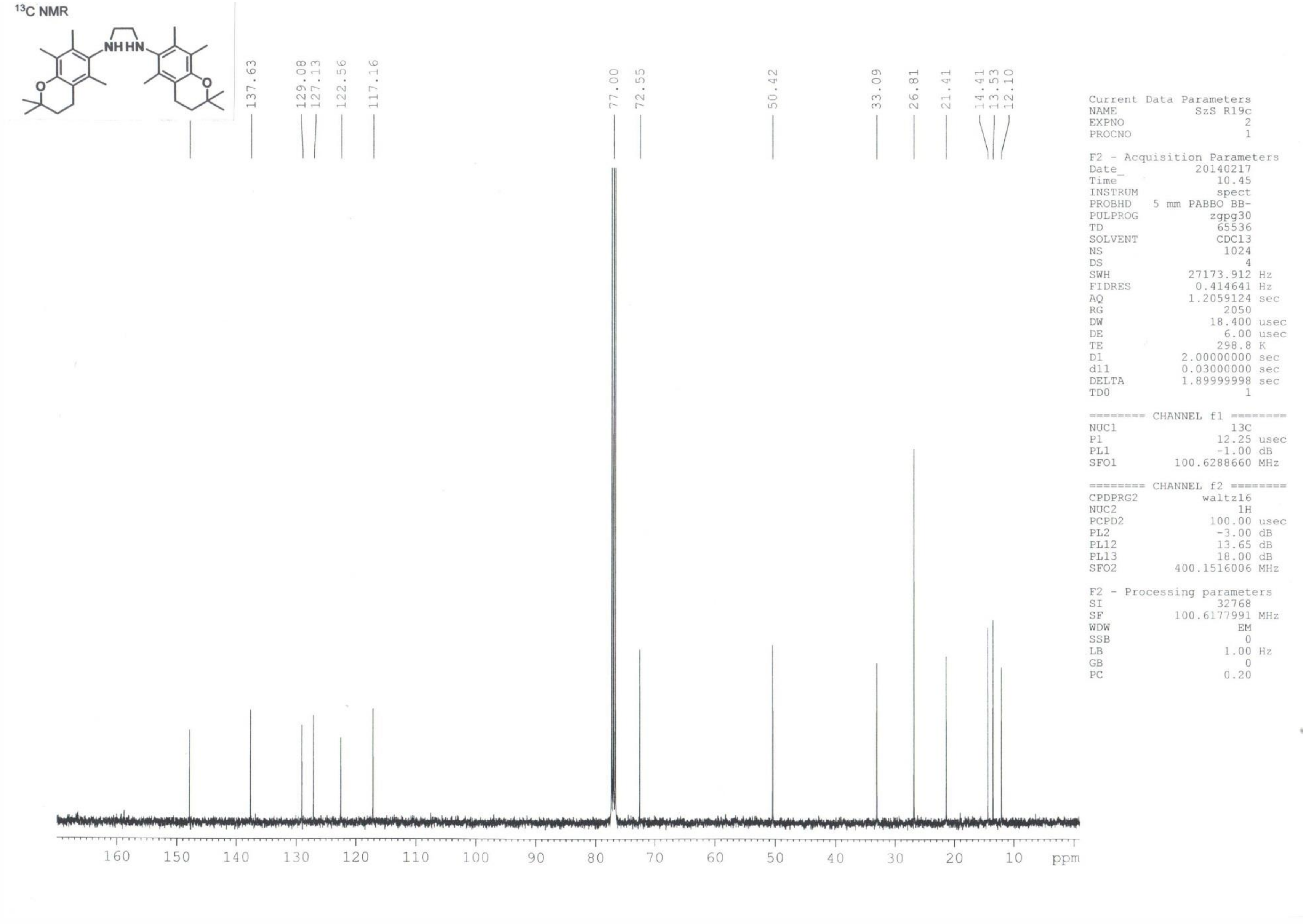

^{1}H NMR spectrum of 1,3-*bis*(2,2,5,7,8-pentamethylchroman-6-yl)-4,5-dihydro-1*H*-imidazol-3-ium chloride (**14**)

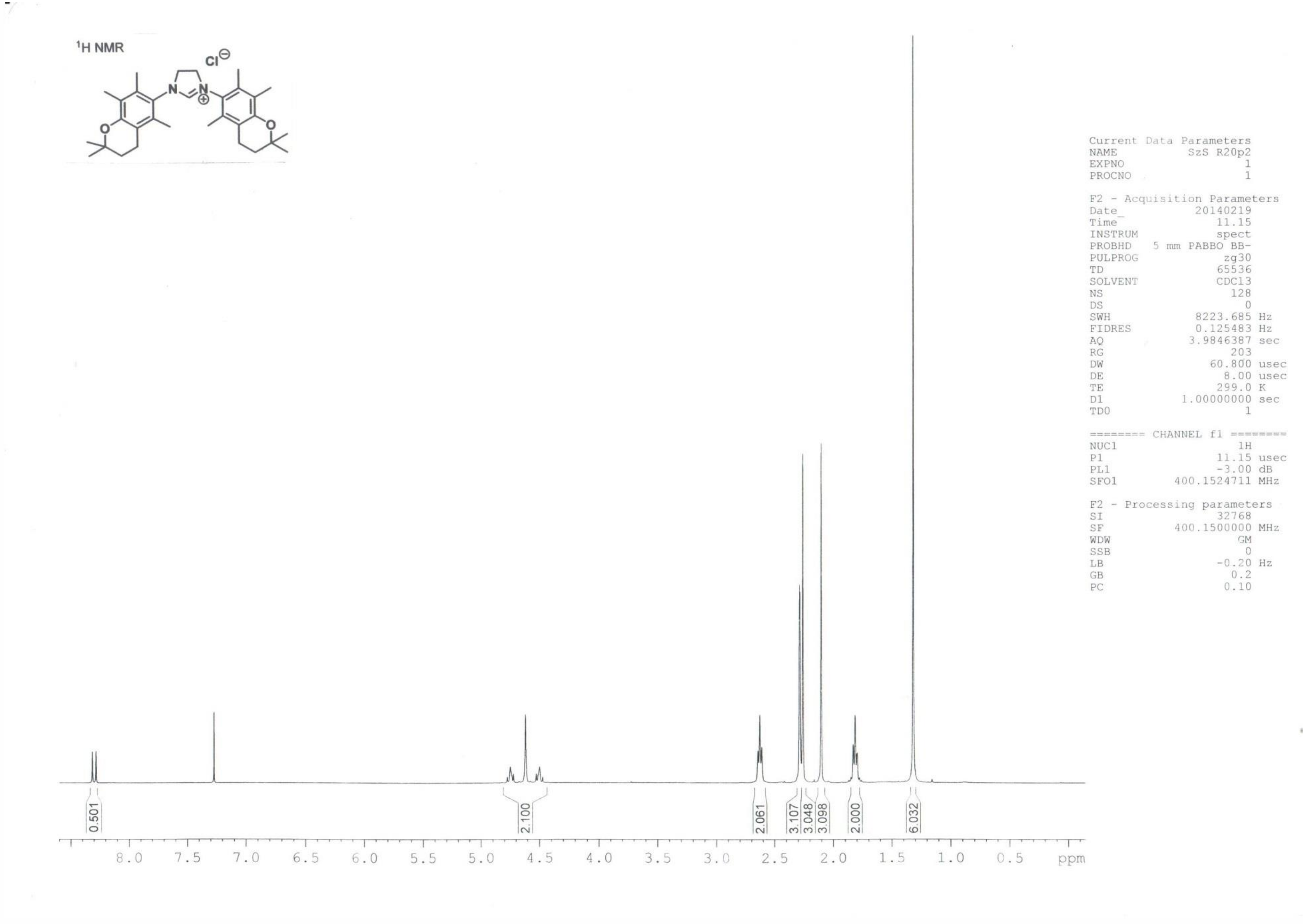

^{13}C NMR spectrum of 1,3-*bis*(2,2,5,7,8-pentamethylchroman-6-yl)-4,5-dihydro-1*H*-imidazol-3-ium chloride (**14**)

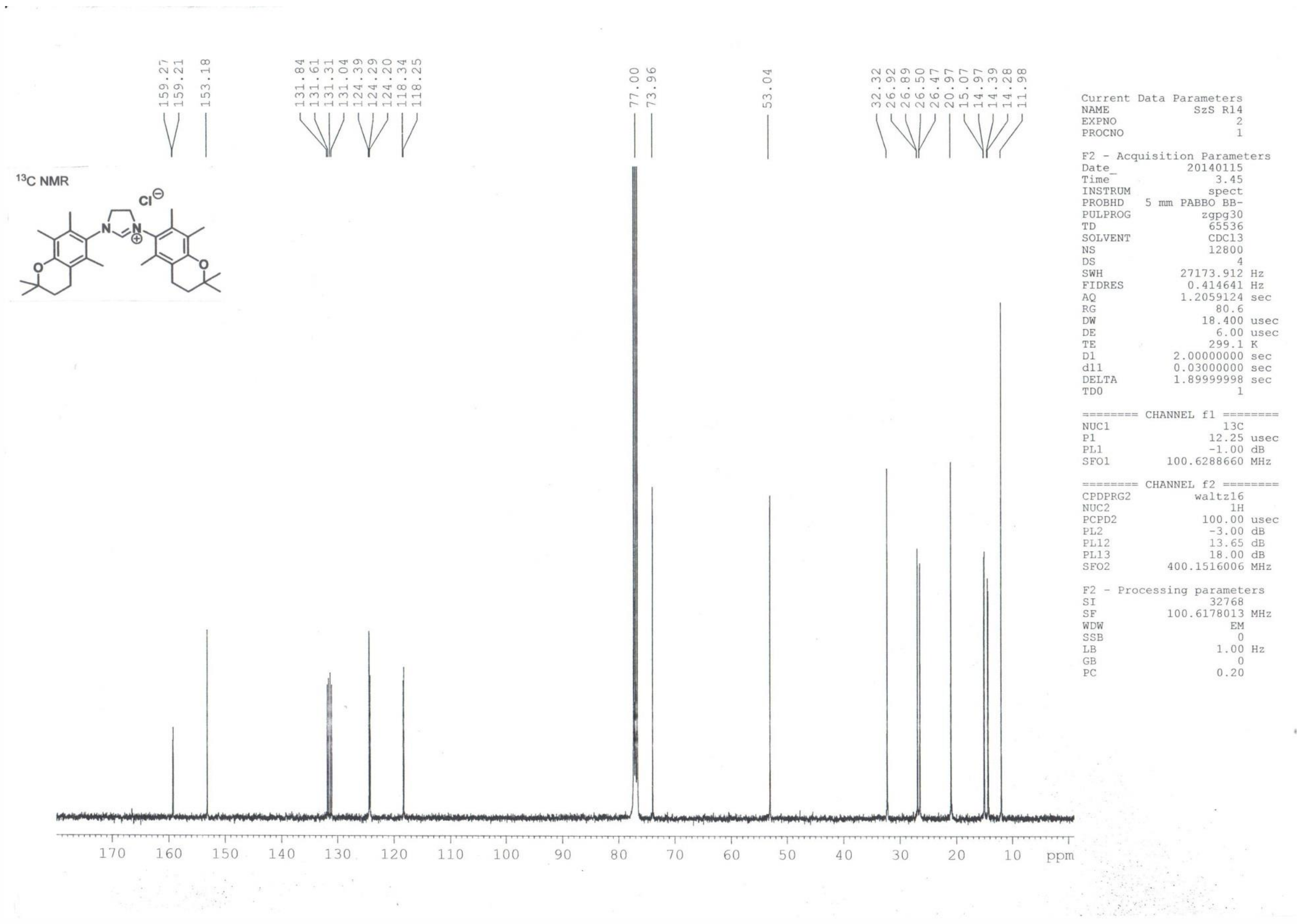

HSQC spectrum of 1,3-*bis*(2,2,5,7,8-pentamethylchroman-6-yl)-4,5-dihydro-1*H*-imidazol-3-ium chloride (**14**)

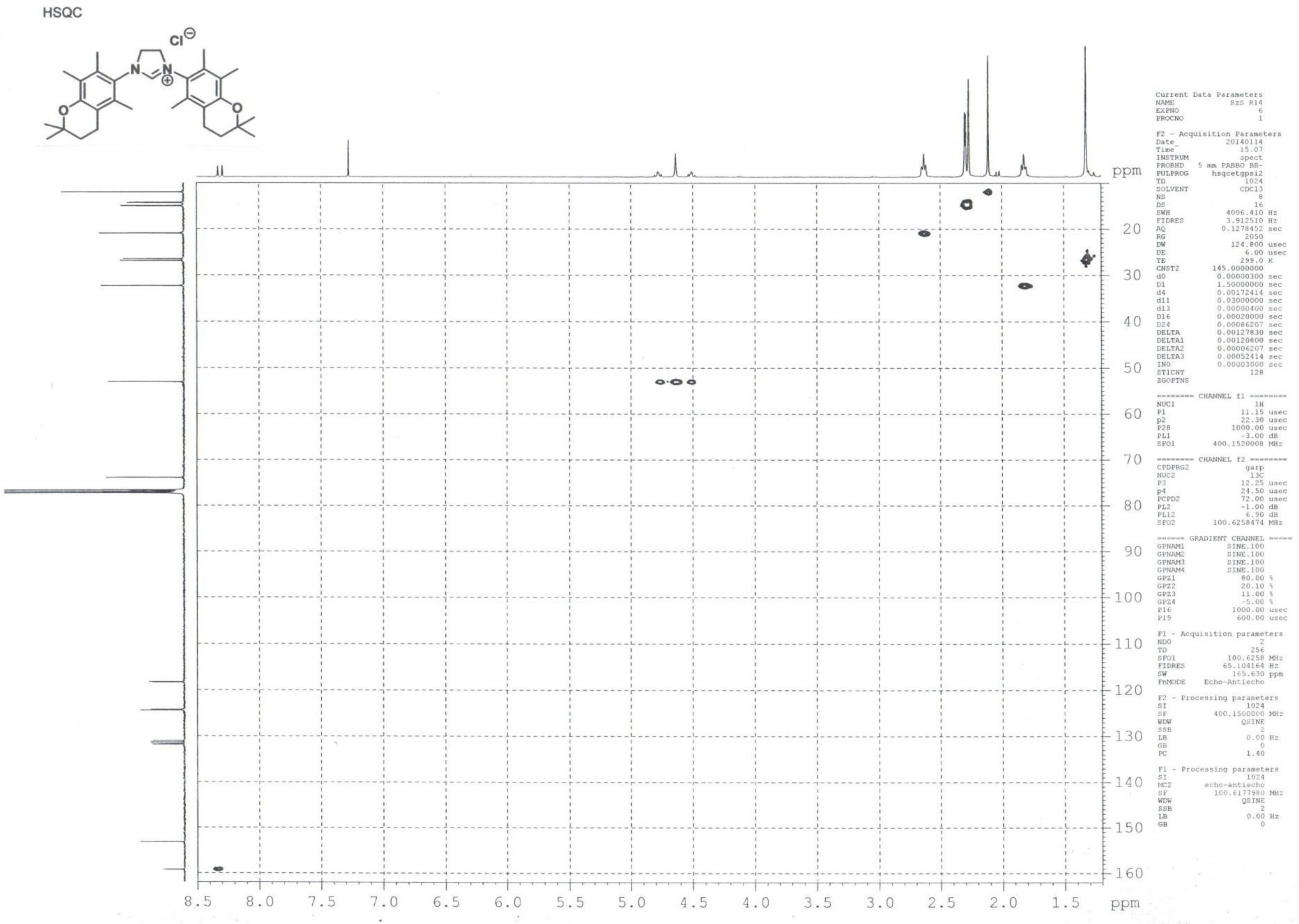

^{1}H NMR spectrum of the catalyst **9**

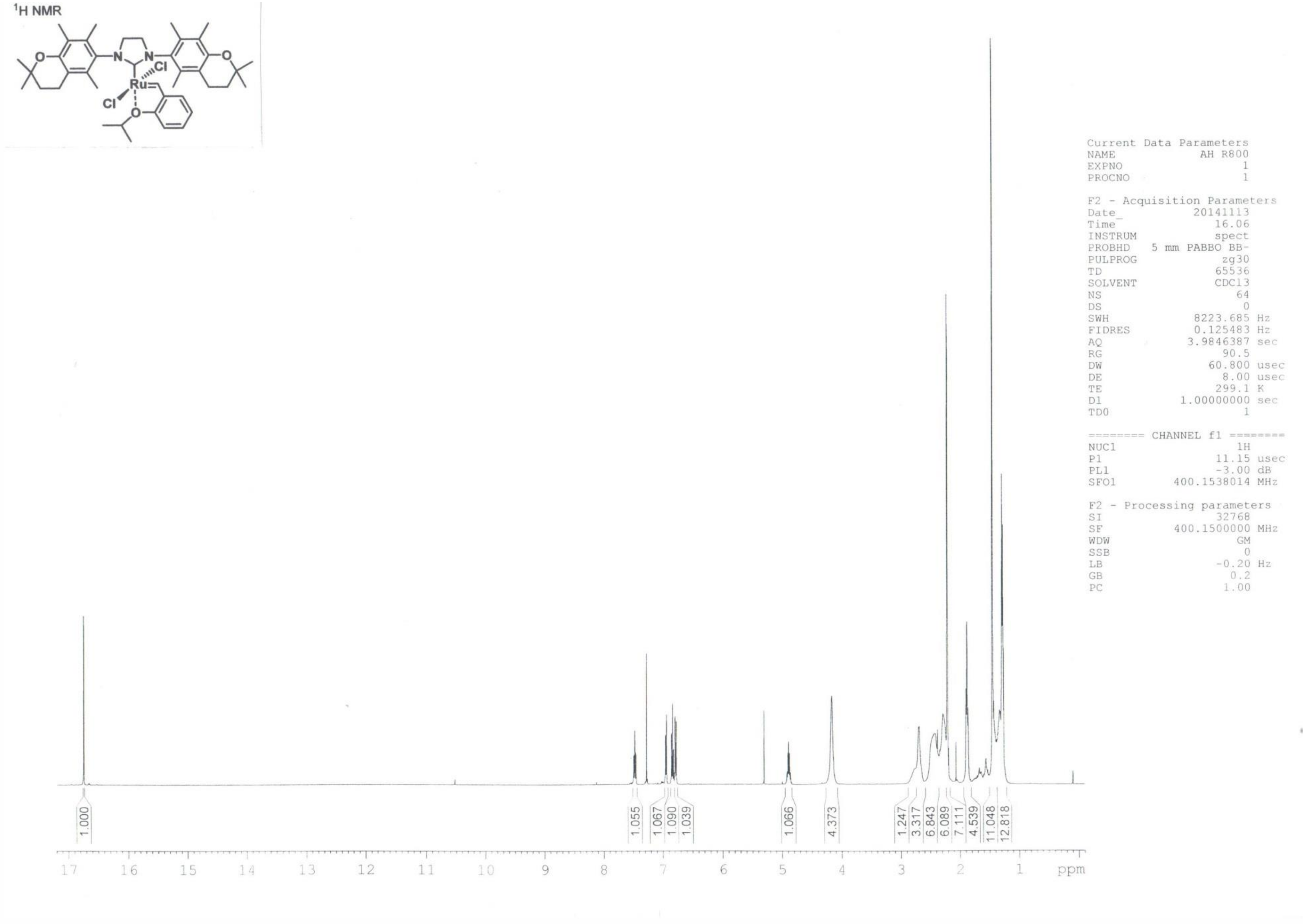

^{13}C NMR spectrum of the catalyst **9**

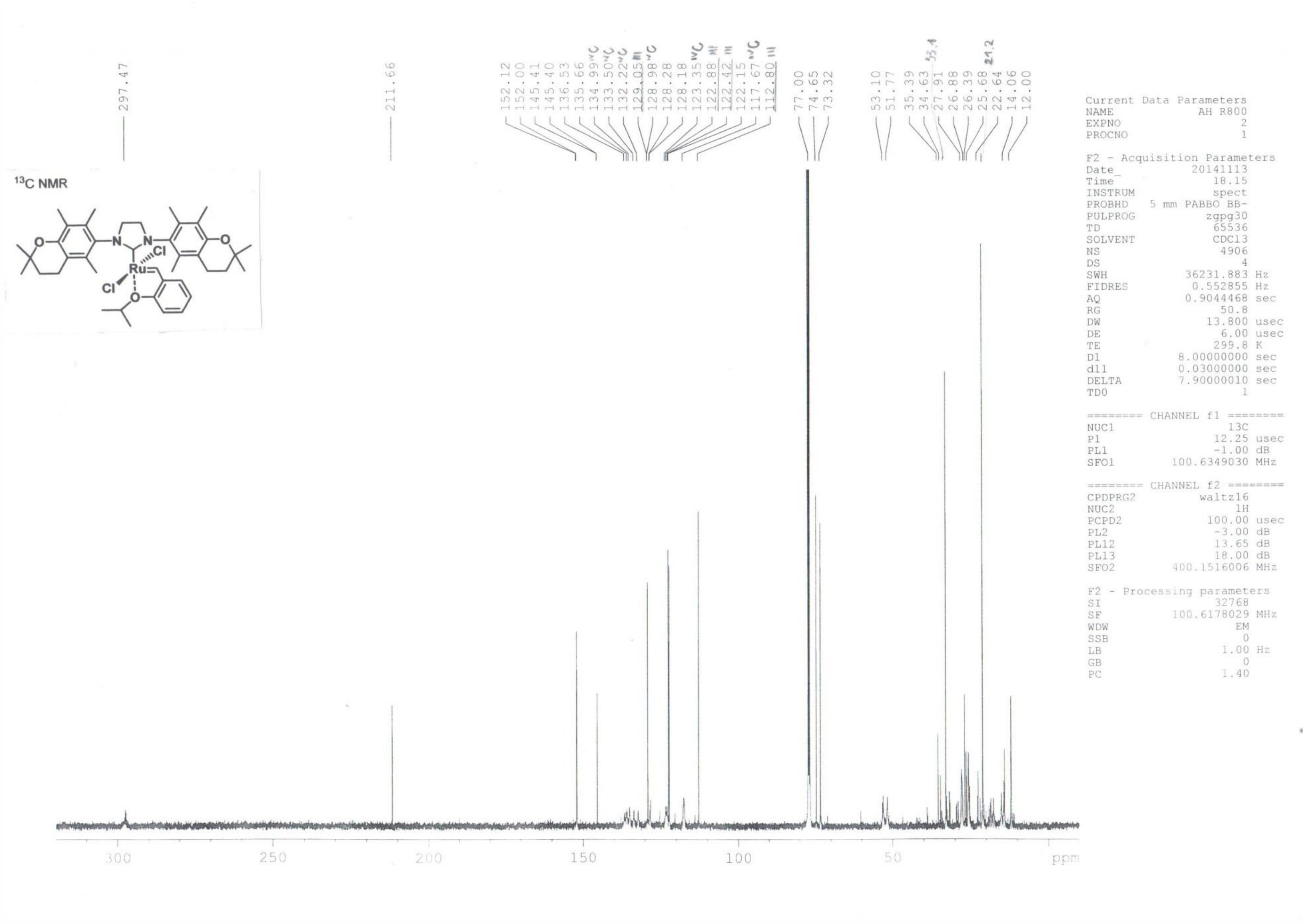

HSQC spectrum of the catalyst **9**

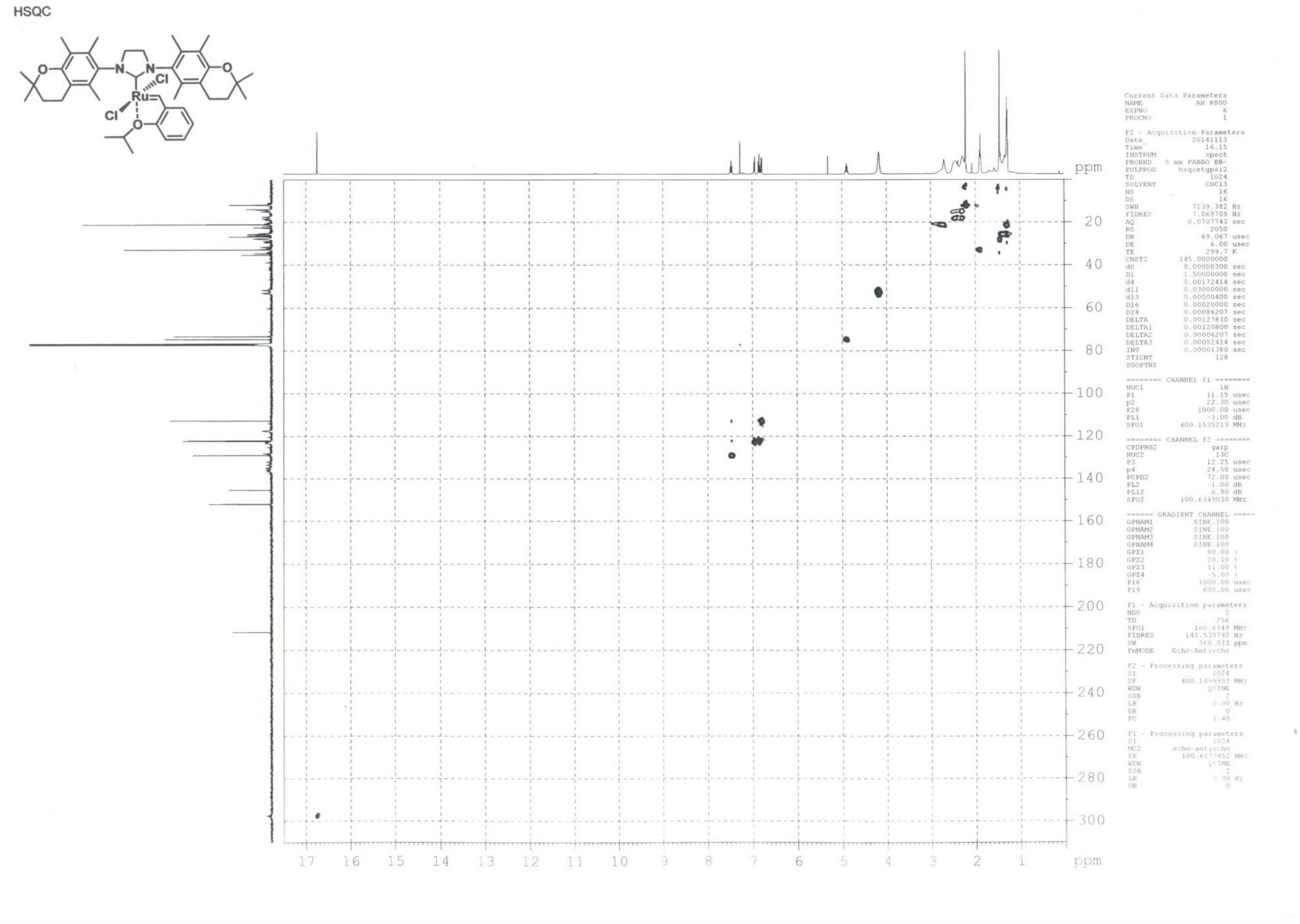

HSQC spectrum of the catalyst **9**

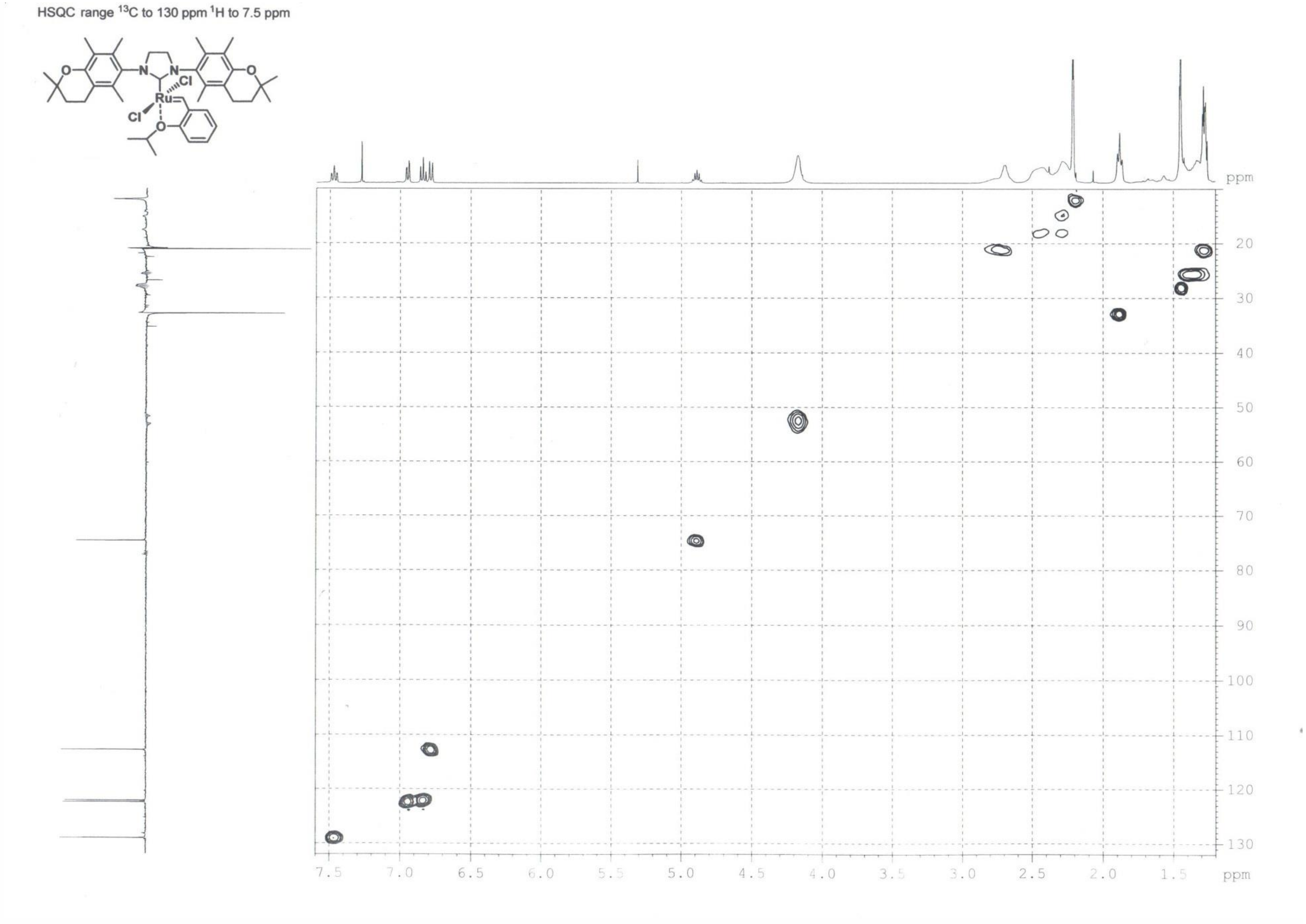

^{1}H NMR spectrum of the catalyst **9**

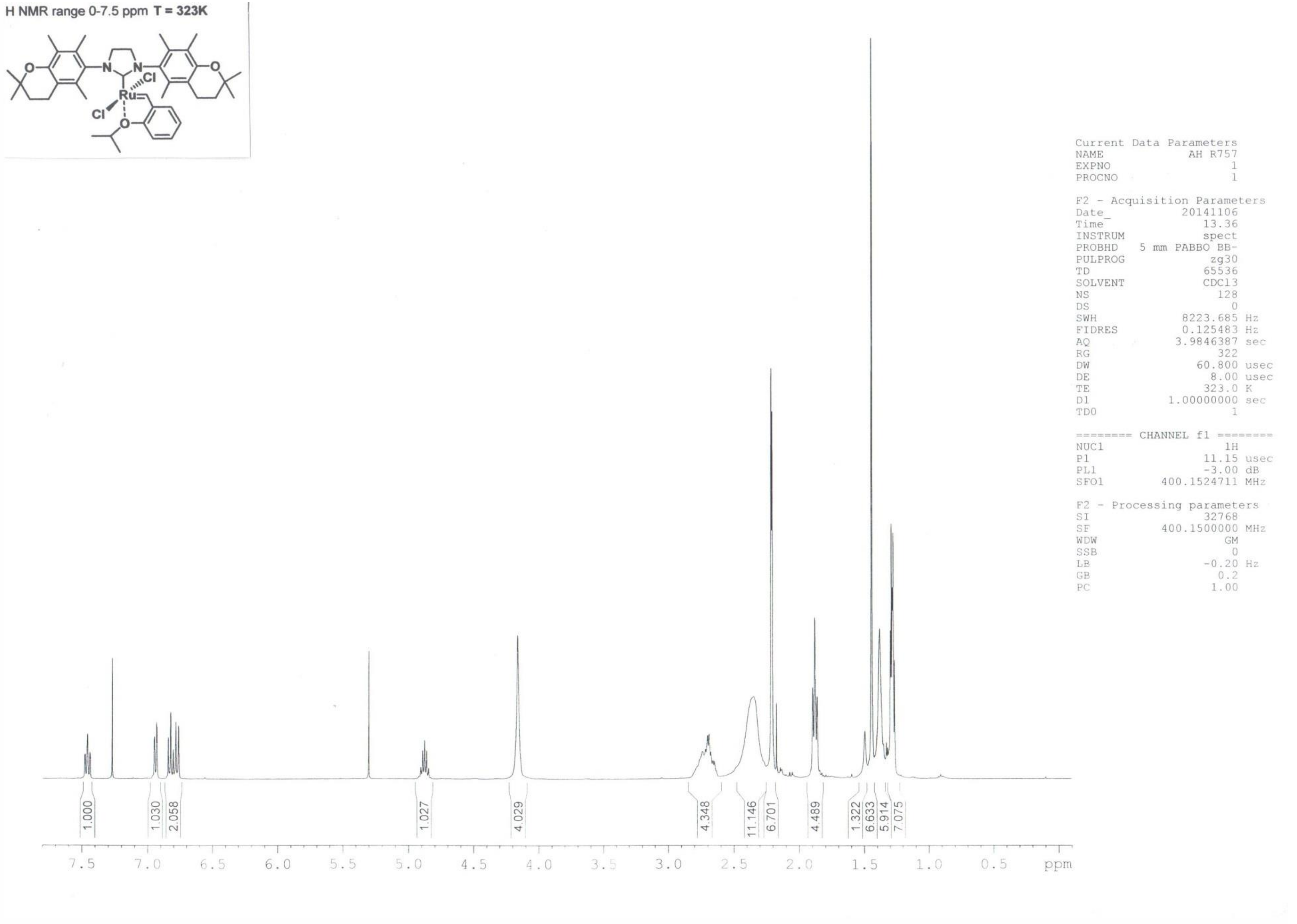

^{13}C NMR spectrum of the catalyst **9**

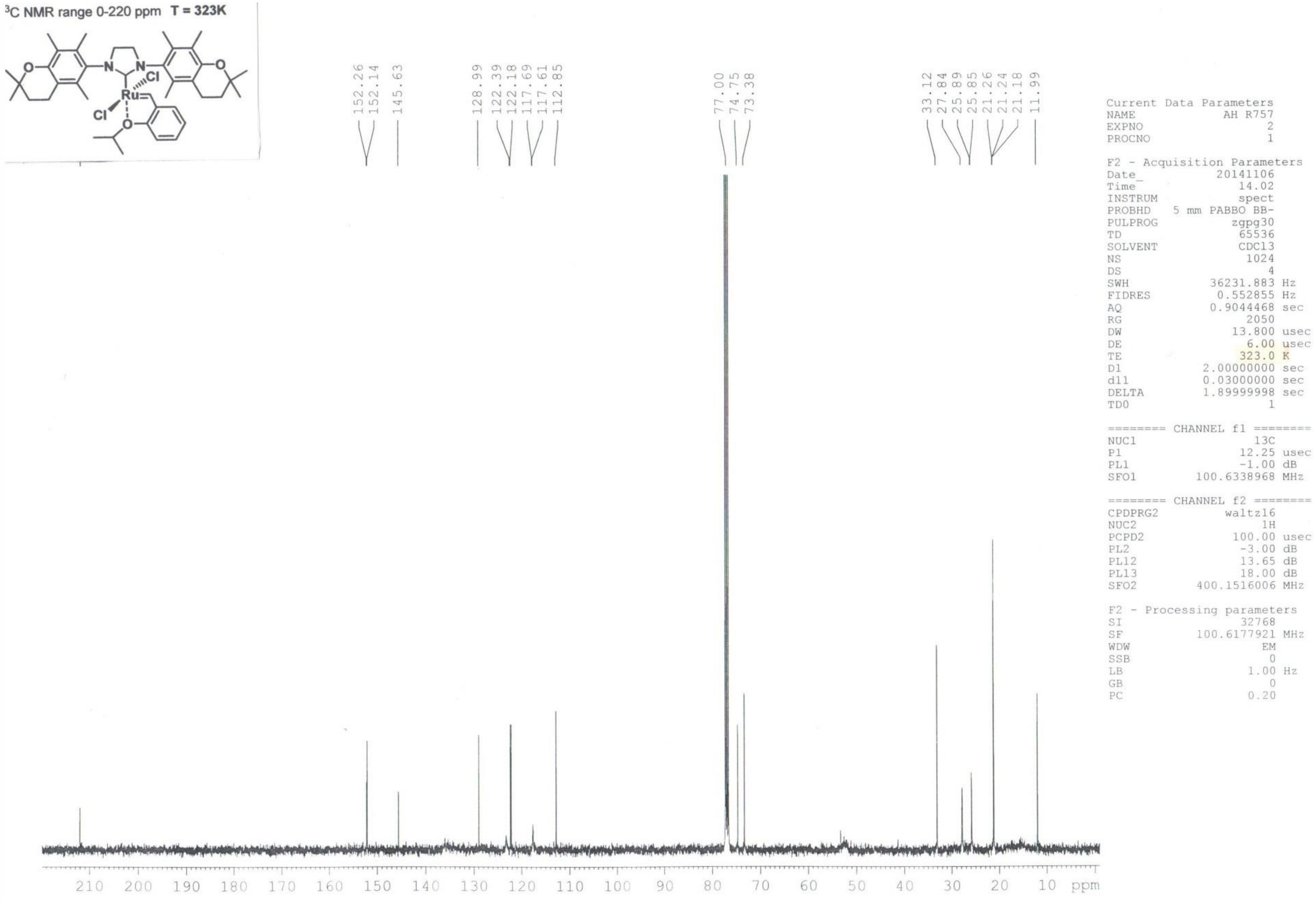

^{13}C DEPT spectrum of the catalyst **9**

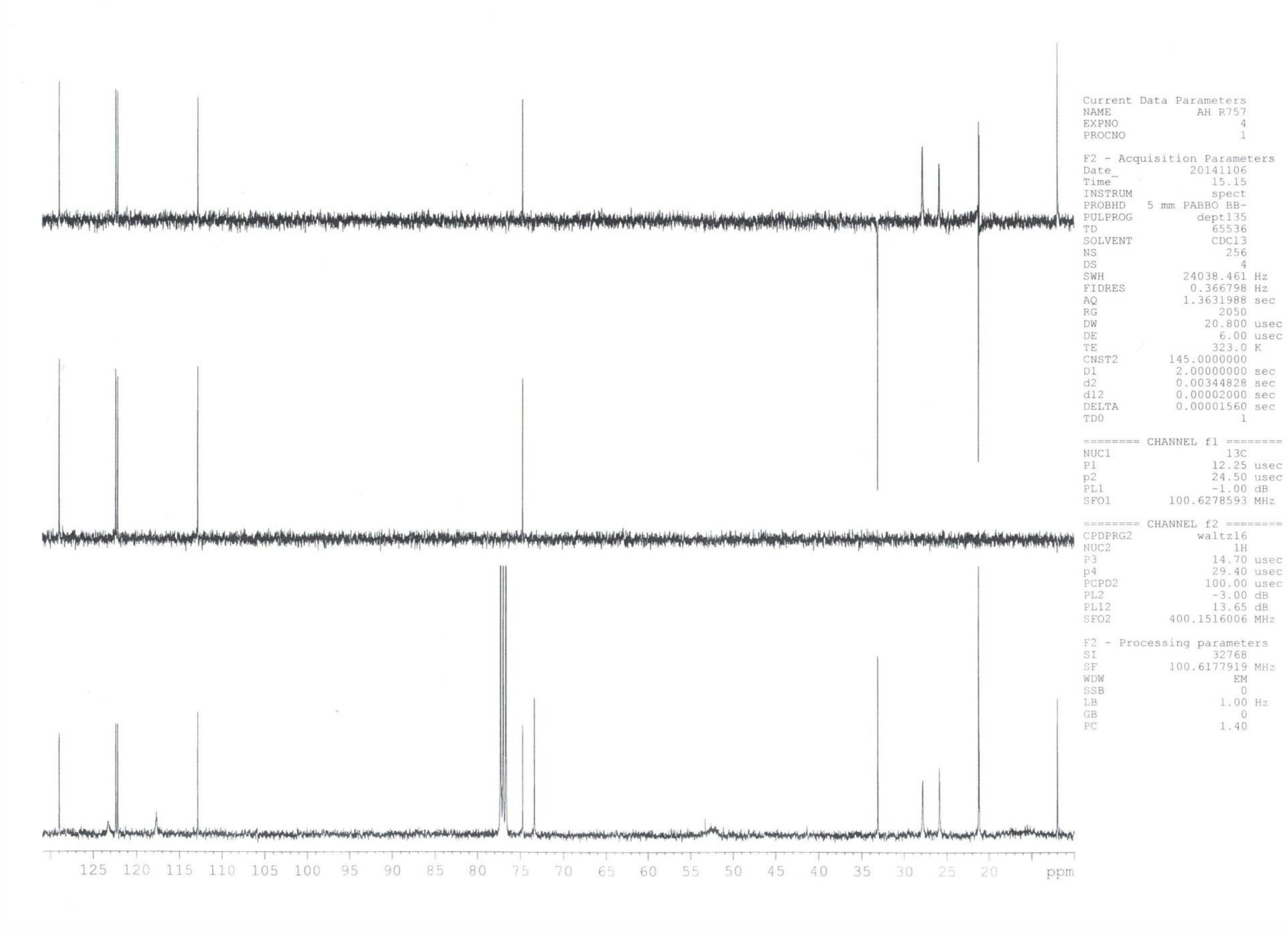

References

1. Bower, J. F.; Race, N. I. *Org. Lett.* **2013,** *15*, 4616-4619.
2. Louie, J.; Bielawski, C. W.; Grubbs, R. H. *J. Am. Chem. Soc.* **2001,** *123*, 11312-11313.
3. Rivard, M.; Blechert, S. *Eur. J. Org. Chem.* **2003**, 2225-2228.
4. Hryniewicka, A.; Misztalewska, I.; Czajkowska-Szczykowska, D.; Urbańczyk-Lipkowska, Z.; Morzycki, J. W.; Witkowski, S. *Tetrahedron* **2014,** *70*, 6810-6816.
5. Taber, D. F.; Frankowski, K. J. *J. Org. Chem.* **2003,** *68*, 6047-6048.
6. Shekhter, O. V.; Sergovskaya, N. L.; Tsizin, Y. S.; Pridantseva, E. A. *J. Org. Chem. USSR (English Translation)* **1982,** *18*, 656-661.
7. Maj, J.; Morzycki, J. W.; Rárová, L.; Wasilewski, G.; Wojtkielewicz, A. *Tetrahedron Lett.* **2012,** *53*, 5430-5433.
8. Wenkert, E.; Bakuzis, M. L. F.; Buckwalter, B. L.; Woodgate, P. D. *Synth. Commun.* **1981,** *11*, 533-544.
9. Gardette, M.; Alexakis, A.; Normant, J. F. *Tetrahedron* **1985,** *41*, 5887-5900.
10. Bunce, R. A.; Bennett, M. J. *Synth. Commun.* **1993,** *23*, 1009-1020.
11. Wojtkielewicz, A.; Maj, J.; Morzycki, J. W. *Tetrahedron Lett.* **2009,** *50*, 4734-4737.
